# Flexible TALEs for an expanded use in gene activation, virulence and scaffold engineering

**DOI:** 10.1093/nar/gkac098

**Published:** 2022-02-12

**Authors:** Sebastian Becker, Stefanie Mücke, Jan Grau, Jens Boch

**Affiliations:** Department of Plant Biotechnology, Institute of Plant Genetics, Leibniz Universität Hannover, 30419 Hannover, Germany; Department of Plant Biotechnology, Institute of Plant Genetics, Leibniz Universität Hannover, 30419 Hannover, Germany; Institute of Computer Science, Martin Luther University Halle-Wittenberg, 06120 Halle (Saale), Germany; Department of Plant Biotechnology, Institute of Plant Genetics, Leibniz Universität Hannover, 30419 Hannover, Germany

## Abstract

Transcription activator-like effectors (TALEs) are bacterial proteins with a programmable DNA-binding domain, which turned them into exceptional tools for biotechnology. TALEs contain a central array of consecutive 34 amino acid long repeats to bind DNA in a simple one-repeat-to-one-nucleotide manner. However, a few naturally occurring aberrant repeat variants break this strict binding mechanism, allowing for the recognition of an additional sequence with a −1 nucleotide frameshift. The limits and implications of this extended TALE binding mode are largely unexplored. Here, we analyse the complete diversity of natural and artificially engineered aberrant repeats for their impact on the DNA binding of TALEs. Surprisingly, TALEs with several aberrant repeats can loop out multiple repeats simultaneously without losing DNA-binding capacity. We also characterized members of the only natural TALE class harbouring two aberrant repeats and confirmed that their target is the major virulence factor *OsSWEET13* from rice. In an aberrant TALE repeat, the position and nature of the amino acid sequence strongly influence its function. We explored the tolerance of TALE repeats towards alterations further and demonstrate that inserts as large as GFP can be tolerated without disrupting DNA binding. This illustrates the extraordinary DNA-binding capacity of TALEs and opens new uses in biotechnology.

## INTRODUCTION

Transcription activator-like effectors (TALEs) are DNA-binding proteins that are well known and widely used for their simple DNA-binding mechanism and their easy reprogrammability. In nature, TALEs are employed by plant pathogenic bacteria of the genus *Xanthomonas*, which use a type III secretion system to inject them directly into plant cells (1). Once inside the plant cell, TALEs localize to the nucleus and search the genome for potential target sites. Upon binding a matching sequence, TALEs upregulate the expression of their downstream target gene(s) (1). In essence, the induction of different target genes aims to support bacterial colonization of the plant (1,2). *Xanthomonas oryzae* pv. *oryzae* (*Xoo*) and *X. oryzae* pv. *oryzicola* (*Xoc*) are severe pathogens of the major food crop rice, infecting the vascular system and the leaf mesophyll, respectively. They contain a large repertoire of TALEs to specifically induce expression of a variety of plant genes (3–5). For *Xoo*, SWEET sugar exporters are key virulence targets that support growth and spreading of the pathogen (6). So far, three different *SWEET* genes have been shown to be induced by *Xoo*, namely *OsSWEET11* (7,8), *OsSWEET13* (6) and *OsSWEET14* (9–12). Plant resistance can be established if the target sequence of such TALEs is mutated either by natural variation (6–8,13–16) or via genome editing (17–21), thus preventing binding of the natural TALE to the altered sequence.

**Figure 1.**
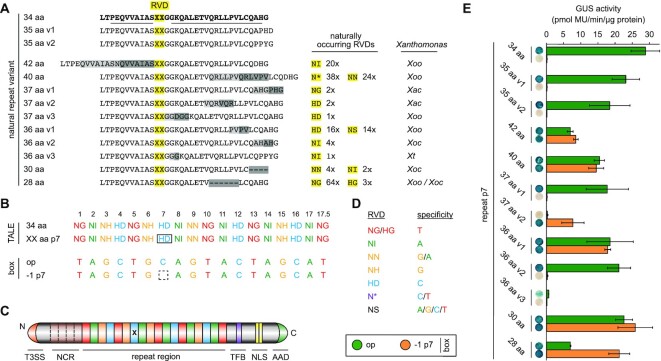
Aberrant repeat variants in *Xanthomonas* TALEs and their impact on DNA binding. (**A**) Amino acid alignment of natural repeat length polymorphisms. A standard 34 amino acid (aa) repeat is shown in bold with helix-forming residues underlined. Duplicated or deleted regions are shaded in grey. *Xanthomonas* *oryzae* pv. *oryzae*, *Xoo*; *X*. *oryzae* pv. *oryzicola*, *Xoc*; *X*. *axonopodis* pv. *citri*, *Xac*; *X. theicola*, *Xt*. (**B**) RVD composition of constructed TALEs. Position 7 (boxed) contains a standard 34 aa repeat (top row) or an aberrant repeat (XX aa) with 42, 40, 37, 36, 35, 30 or 28 aa. The TALE box (optimal, op, or with deletion at position 7, −1 p7) is fused to a minimal promoter and a β-glucuronidase (GUS) reporter gene. (**C**) TALE with type III secretion signal (T3SS), four noncanonical repeats (NCR), TFIIAγ binding site (TFB), nuclear localization signals (NLS) and acidic activation domain (AAD). The x indicates the aberrant repeat. (**D**) DNA specificities of selected RVDs. (**E**) *In planta* GUS assay for aberrant repeat-containing TALEs on boxes shown in panel (B). Error bars represent standard deviation (*n* = 3).

TALEs harbour an N-terminal signal for type III-dependent secretion, two nuclear localization signals, an interaction surface for transcription factor binding, a C-terminal activation domain and a central DNA-binding domain composed of a varying number of highly conserved repeats (Figure 1) (1,22). The unique DNA-binding mechanism of TALEs is governed by a simple code in which one repeat confers binding to exactly one nucleotide of the DNA target sequence (23,24). Binding specificity is mediated by two highly variable residues at positions 12 and 13 within each repeat, termed repeat-variable di-residues (RVDs). Depending on the RVD, each repeat can either specify exactly one nucleotide or allow for a number of different ones—ranging from two or three to all four (Figure 1D) (23,25,26). In addition to the RVDs, a degenerated repeat in the N-terminal region of a TALE specifies an additional T in the target sequence. TALE proteins wrap around the DNA double helix in a right-handed superhelix built from left-handed alpha-solenoid repeats (27,28). Contact to a single base in the leading strand is made via the 13th amino acid of each repeat (27,28). During search mode along a DNA double strand, the TALE protein has an extended, partly relaxed conformation where the N-terminal domain exerts the general DNA-binding activity and linear movement along the DNA strand occurs in a nonrotational fashion (29,30). Upon binding to a target sequence, the repeats condense onto the DNA bases, enabling additional contacts between the positively charged amino acids in each repeat and the negatively charged phosphate backbone of the DNA (27,28).

TALEs have gathered considerable interest, because the repeats can be assembled in any given order to generate any desired DNA-binding specificity for biotechnological applications (31). The most successful application is the use of TALE–FokI nuclease fusions (TALEN) for genome editing (32–34). However, TALEs have also been employed as designer gene activators and repressors, as fusions to recombinases and transposases to trigger targeted recombination events or as fusions to fluorescent proteins for the labelling of repetitive genomic locations (31). Most recently, fusions between TALEs and the bacterial toxin DddA yielded highly efficient TALE base editors that function not only in the nucleus of cells but also in chloroplasts and mitochondria (35–37).

While the vast majority of TALE repeats contain 34 aa, two common exceptions with 33 and 35 aa exist. The 33 aa repeat lacks the amino acid at position 13 and can be found in TALEs throughout all TALE-carrying *Xanthomonas* strains. This RVD is labelled as X* (X being most frequently the amino acid N or H), with the asterisk indicating that this amino acid is missing. Repeats with 35 aa often carry an additional proline at position 33 of the repeat, but other amino acid changes can occur as well. Despite the amino acid differences of those variants, they confer a normal DNA-binding mode (23,38,39). However, rare repeat variants with more profound differences in length and effect have been found in nature as well. As a result of either small duplications or deletions, those so-called aberrant repeats consist of 42, 40, 36, 30 or 28 aa, respectively (3,14,40,41). Some of these aberrant repeats strongly affect the very regular TALE–DNA interaction by enabling a TALE to bind an additional sequence containing a single nucleotide deletion at the position of the aberrant repeat (14). It is believed that those aberrant repeats can loop out of the array and away from the DNA if a matching frameshift sequence is encountered (14). Because the aberrant repeat can either stay in or loop out of the repeat array, TALEs containing such a repeat can flexibly recognize two DNA sequences that differ by one base in length. This is not possible for TALEs containing only standard 34 aa repeats. So far, it is not understood which changes in TALE repeats cause the flexible behaviour of aberrant repeats. In general, TALEs have to fold into their explicitly regular structure to match the regular shape of a DNA double helix. Accordingly, any repeat alterations can pose significant problems to the DNA-binding capacity of TALEs. Exploring the impact of repeat alterations can contribute significantly to our understanding of the TALE protein–DNA interaction.

In this study, we analyse how the naturally occurring aberrant repeats influence TALE DNA binding. Some facilitate flexible frameshift binding, while others remain in or loop out of the repeat array, constitutively. Surprisingly, multiple aberrant repeats can loop out simultaneously and drag other repeats out of the array. We analysed which effect the position and composition of changes within a repeat have on TALE activity. Eventually, we were able to insert a GFP protein into the repeat array while conserving TALE DNA binding. We confirm *OsSWEET13* as the virulence target of the only natural TALE class containing two aberrant repeats and demonstrate that the rice cultivar Sadu Cho shows resistance to all known TALEs.

## MATERIALS AND METHODS

### Bacterial growth conditions

*Escherichia coli* strain Top10 (New England Biolabs) was grown at 37°C using LB media. *Agrobacterium tumefaciens* strain GV3101 was cultivated at 28°C using YEB media, while *Xoo* strains BAI3 and BAI3Δ*talC* and their derivatives were grown at 28°C using PSA media.

### Plant growth conditions

*Nicotiana benthamiana* plants were grown under greenhouse conditions with 16 h of light, relative humidity of 40–60% and temperatures of 23 and 19°C, during daytime and night, respectively. After growing for 4–6 weeks, plants were used for inoculation experiments. *Oryza sativa* plants from the cultivars Zhenshan, Kitaake, Sadu Cho and IR24 were grown under greenhouse conditions with a relative humidity of 70% and temperatures of 28 and 25°C, during daytime and night, respectively. After 3–5 weeks of growth, plants were used for inoculation experiments.

### Identifying TALE repeats of aberrant length

In order to identify novel aberrant repeat length variants, all available TALE-carrying *Xanthomonas* genomes as well as all available individual TALE sequences were analysed. For this, the AnnoTALE ‘TALE prediction’ and ‘TALE analysis’ functions (42) were combined with a manual evaluation process. All *Xanthomonas* repeats with <33 or >35 aa were classified as aberrant repeats.

### Construction of novel aberrant repeat modules and artificial TALEs

In order to construct novel aberrant repeat modules, the required amino acid changes were introduced into existing repeat modules of standard length via PCR. Oligonucleotides are listed in Supplementary Table S1. Artificial TALEs were generated using the Golden TAL Technology assembly kit (43). In the first step, up to six single repeats were combined into one of the available assembly vectors. In the second assembly step, several of these multi-repeat modules were combined with the N- and C-terminal regions of a TALE and the desired expression vector to obtain the final TALE expression constructs. The N- and C-terminal regions were derived either from Hax3 (44) or from TalAG4 and TalAO3 from *Xoo* strain PXO83 (5). For the *A. tumefaciens*-mediated inoculation of *N. benthamiana* and the *Xanthomonas*-mediated inoculation of rice, the expression vectors pSKA2 and pSKX1 were used, respectively.

### Reporter constructs and GUS analysis

To generate GUS reporter constructs, PCR-amplified promoter fragments from rice or artificial TALE target boxes in combination with the *Bs4* minimal promoter were placed in front of a promoterless *uidA* reporter gene by inserting them into the vector pGWB3GG, a Golden Gate-compatible variant of pGWB3. Oligonucleotides are listed in Supplementary Table S1.

### *Nicotiana benthamiana* infiltration and GUS reporter assay

*Agrobacterium tumefaciens* strains containing the reporter- and TALE-expressing constructs were resuspended in *Agrobacterium* infiltration media and adjusted to an OD_600_ of 0.8. For the inoculation of *N. benthamiana* plants, one reporter- and one TALE-expressing construct were mixed in a 1:1 ratio and then infiltrated using a needleless syringe. Two days after inoculation, two leaf discs (diameter 0.9 cm) were harvested from each inoculation spot and frozen via liquid nitrogen. The samples were stored at −80°C until further use. All GUS analyses were performed as previously described (23). Experiments were repeated at least twice.

### *Oryza sativa* virulence assay

The two youngest leaves of 3–5-week-old plants were inoculated with *X. oryzae* strains BAI3 and BAI3Δ*talC* or one of their derivatives using a needleless syringe. Prior to the inoculation, the bacterial suspensions were adjusted to an OD_600_ of 0.5 using 10 mM MgCl_2_. Each strain was inoculated on three different rice plants, using two leaves per plant by placing three to four spots within the first 5 cm of each leaf tip. Five days after inoculation, leaves were harvested and symptoms were documented.

### Prediction of TALE target genes

TalBK2 target genes were predicted using the ‘Predict and Intersect Targets’ tool of AnnoTALE version 1.4.1 (42,45). For this analysis, promoter sequences from 300 bp upstream to 200 bp downstream of the transcriptional start site or the start codon were utilized. The promoter sequences were extracted from the *O. sativa* genome MSU7.

## RESULTS

### Naturally occurring aberrant repeats

To understand the variety of natural length polymorphisms in TALE repeats, we analysed all available TALE sequences from *Xanthomonas* spp. All repeats that deviate in length from standard repeats (34 aa) or from commonly occurring length variants (33 and 35 aa) were classified as aberrant repeats. Within 276 completely sequenced TALE-containing *Xanthomonas* genomes and multiple individual TALE sequences, we identified 195 repeats of aberrant length in 180 TALEs with 77 unique RVD sequences. One hundred sixty-five TALEs carry a single and 15 TALEs carry two aberrant repeats (Supplementary Figure S1; Supplementary Tables S2 and S3). Sixty-seven TALEs share small N- and C-terminal deletions that mark them as part of the group of truncated TALEs (truncTALEs)/interfering TALEs (iTALEs) (40,41), while four other TALEs harbour larger deletions and are annotated as putative pseudogenes. Most TALEs with aberrant repeats are from Asian *Xoo* or *Xoc* strains with four exceptions identified in *X. axonopodis* pv. *citri* and one in the *X. theicola* strain CFBP4691. Every completely sequenced Asian *X. oryzae* strain carries at least one TALE with an aberrant repeat (Supplementary Figures S1 and S2). According to the AnnoTALE nomenclature (42), the aberrant repeat-carrying TALEs are categorized into 24 different TALE classes (Supplementary Figures S1 and S2; Supplementary Table S2). The 195 aberrant repeats themselves can be categorized into 10 different length variants with 42, 40, 37 (versions 1, 2 and 3), 36 (versions 1, 2 and 3), 30 and 28 aa, respectively (Figure 1A; Supplementary Tables S2 and S3). For repeat variants with 42, 40 and 30 aa, it was shown earlier that they mediate a flexible recognition of a perfectly matching sequence and a −1 nucleotide frameshift sequence (14), while an obligatory frameshift recognition has been described for the 28 aa variant (41). In contrast, none of the other variants have been analysed so far.

### Not all aberrant repeats facilitate a flexible frameshift recognition

To analyse the impact of the novel aberrant repeats on the DNA-binding mode of TALEs, we introduced a single aberrant repeat of each type into an artificial TALE. To build artificial TALEs, all aberrant repeat variants were constructed as modules (Supplementary Table S4) for our Golden TAL Technology assembly kit (43). The TALEs contained 17.5 repeats with an alternating DNA-binding specificity, to maximize frameshift impact. As control, a TALE containing only 34 aa standard repeats was used. We also included two different TALE repeats with 35 aa since they were never analysed for their ability to confer frameshift tolerance. All aberrant repeats were placed at the same position (position 7) and contained the same RVD (HD) (Figure 1A–D). The TALEs were tested in combination with two different target TALE boxes in a GUS reporter assay. One TALE box was the optimal sequence based on the RVDs (op), and the other one was a frameshift derivative with a single nucleotide deleted at the position corresponding to the aberrant repeat (−1 p7). If a TALE successfully binds to the target box, the GUS reporter gene is induced and activity can be detected.

The control TALE with only 34 aa repeats can activate transcription at the optimal box but is unable to address the frameshift derivative (Figure 1E). Similar observations were made for the two repeat variants with 35 aa, the variant 36 aa v2 and the variant 37 aa v1, making the latter two the first natural aberrant repeats unable to confer a frameshift recognition (Figure 1E). In contrast, the TALEs containing one of the aberrant repeats with the known ability to confer frameshift recognition (30, 40 and 42 aa) and the variant 36 aa v1 are able to flexibly address both boxes (Figure 1E). The TALE containing the 28 aa repeat shows a clear and reproducible preference for the frameshift box while still being able to address the optimal box. This observation partially contradicts earlier reports in which the 28 aa variant conferred obligatory frameshift binding (41). The TALE containing the newly identified aberrant repeat 37 aa v2, however, showed activity only on the frameshift box, rendering it the only natural repeat variant that loops out constitutively (Figure 1E). The last novel aberrant repeat, the variant 36 aa v3, is based on a repeat with 35 aa. While it contains only a single amino acid duplication, this duplication, an additional G, is placed right after the RVD, and thus at a position with crucial impact on repeat functionality. As a consequence, the activity of a TALE with even a single 36 aa v3 repeat is barely detectable (Figure 1E).

We can thus categorize aberrant repeats also based on their functionality. The first category does not affect TALE binding, the second one facilitates a facultative frameshift recognition, the third is completely unable to participate in DNA recognition, likely by looping out of the repeat array, while the fourth and last category impairs overall TALE function.

### The novel aberrant repeats themselves loop out

TALEs with an aberrant repeat of 42, 40 or 30 aa show the highest activity on a TALE box with either no deletion or a deletion exactly corresponding to the position of the aberrant repeat (14). This leads to the conclusion that the aberrant repeats loop out themselves when challenged with a frameshift sequence, instead of forcing out one of the adjacent repeats. Based on these data, we wanted to clarify how the novel identified aberrant repeats with 37, 36 or 28 aa behave. To answer this question, we tested our TALEs with the various aberrant repeats at position 7, as well as our control TALE with exclusively 34 aa repeats, on TALE boxes with a one-nucleotide deletion at position 6, 7 or 8 (Supplementary Figure S3A–D). All TALEs containing an aberrant repeat that is able to facilitate a facultative frameshift recognition showed highest activity either on an optimal box or on a box with a −1 nucleotide deletion corresponding exactly to the aberrant repeat (Supplementary Figure S3F and G). Furthermore, the TALE containing the variant 37 aa v2 could only activate the box with a −1 deletion at position 7, supporting our previous observation that this repeat variant loops out constitutively. In contrast, TALEs with 37 aa v1, 36 aa v2 or 35 aa behaved exactly like a TALE solely constructed with standard 34 aa repeats and recognized only the optimal sequence (Supplementary Figure S3F and G). These results show that all aberrant repeats that confer recognition of a frameshift sequence prefer to loop out of the repeat array themselves.

We further investigated whether aberrant repeats can mediate binding to a −2 nucleotide frameshift sequence, for example by causing disturbances in the stabilizing inter- and intra-repeat interactions so that one of the adjacent normal repeats is forced out together with them. For this, we used target boxes containing a −2 nucleotide deletion at the positions 6 and 7 or 7 and 8 (Supplementary Figure S3E) and tested them together with TALEs harbouring one of the natural aberrant repeats at position 7. In contrast to a 34 aa standard repeat, all aberrant repeats with the ability to confer a −1 nucleotide frameshift recognition were also able to allow for the recognition of a −2 frameshift box; however, the observed activity was extremely low (Supplementary Figure S3H and I). Nonetheless, this demonstrates that aberrant repeats do have a weakening effect on the inter- and intra-repeat interactions of a TALE and that this effect can lead to multiple repeats being excluded from the DNA binding simultaneously. This observation is the first indication that a TALE is able to loop out multiple repeats to fit to a target sequence (Supplementary Figure S3J).

### Multiple aberrant repeats in tandem can loop out at once

**Figure 2.**
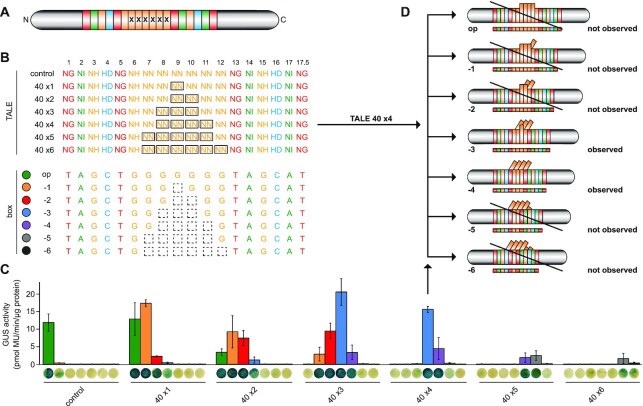
TALEs with multiple aberrant repeats in tandem loop out multiple repeats. (**A**) TALE set-up. Altered repeats are indicated by x. (**B**) RVDs of constructed TALEs containing zero (control) or one to six aberrant 40 aa repeats (boxed RVDs). TALE boxes were optimal (op) or frameshift variants with one to six nucleotides deleted (−1 to −6; dashed squares). (**C**) GUS assay of TALEs on boxes shown in panel (B). Error bars represent standard deviation (*n* = 3). Colours refer to the boxes in panel (B). (**D**) Possible binding behaviour of the TALE with four aberrant repeats (longer rectangles). Activity was observed only if combined with boxes that had three or four nucleotides deleted (−3 or −4), suggesting a simultaneous looping out of multiple aberrant repeats (tilted rectangles).

Based on the observation that an artificial TALE with two 40 aa aberrant repeats in tandem showed activity in our previous study (14), we wondered whether it is possible to create a functional TALE with multiple aberrant repeats. We therefore generated TALEs with zero to six of the 40 aa aberrant repeats placed in tandem. Those TALEs were tested on an optimal box and on target sequences that had one to six nucleotides deleted corresponding to the positions of the aberrant repeats (Figure 2A and B). We wanted to limit the number of necessary boxes to a minimum, while on the other hand gain full readout on how many aberrant repeats are tolerated and how many repeats can loop out. Therefore, we designed the centre of the repeat region (repeats 7–12) containing only NN repeats, which can bind to any of the Gs in the target sequence, while placing six repeats with mixed RVDs as ‘specific anchors’ in the beginning and the end of the TALEs to enforce an exact positioning of both TALE ends (Figure 2A and B). With this set-up, we were able to test all TALEs on the same set of reporter boxes. This experiment demonstrated that a TALE can in principle tolerate multiple aberrant repeats (Figure 2C). In our set-up, a TALE with as many as four aberrant repeats in tandem still showed full activity (Figure 2D), while the activity of TALEs with more aberrant repeats dropped significantly (Figure 2C). Since TALEs with multiple aberrant repeats yielded only high activities on boxes with several nucleotides deleted, we conclude that multiple aberrant repeats in tandem tend to loop out simultaneously (Figure 2C and D). This offers further insight into the structure and function of TALEs, indicating that aberrant repeats weaken the repeat-to-repeat interaction such that a looping out is the preferred conformation. At the same time, it appears that at least four repeats looping out at once is tolerated without penalty for the overall activity, possibly, because the remaining repeats have enough binding affinity to keep the TALE connected to the DNA target sequence.

### Neighbourhood community: the nature of an aberrant repeat controls its impact on either flanking repeats

By comparing TALEs containing two or three aberrant repeats in tandem of either 40 or 42 aa length, we observed that they do not activate the same reporter constructs (Supplementary Figure S4). Based on this observation, we generated TALEs containing each aberrant repeat placed at positions 7 (HD) and 8 (NN) (Supplementary Figure S5). We used for each of the two aberrant repeats RVDs with a different nucleotide specificity to be able to distinguish which of the two aberrant repeats participates in DNA binding and which loops out. We tested the TALEs on four different target boxes, with no deletion (op), the nucleotide deleted that corresponds to the first aberrant repeat (−1 p7), the nucleotide deleted that corresponds to the second aberrant repeat (−1 p8) or both nucleotides deleted (−2). For TALEs with two 28 and 37 aa v2 repeats, respectively, we saw exclusive activity on the −2 box, showing that both always loop out simultaneously (Supplementary Figure S5). In contrast, the TALE with the two 30 aa repeats in tandem activated only the optimal and the −1 p8 box, indicating that the first aberrant repeat cannot loop out. The 42, 40 and 36 aa v1 repeats showed activity on −1 and −2 boxes (Supplementary Figure S5). However, which of the two different −1 boxes was activated depended on the aberrant repeat variant present. The TALEs with two 40 or two 36 aa v1 repeats looped out the second or both aberrant repeats, whereas the TALE with two 42 aa repeats looped out the first or both aberrant repeats (Supplementary Figure S5). This suggests that the particular alteration in an aberrant repeat affects its interaction with the repeat either preceding or following the aberrant repeat, and its looping-out behaviour, accordingly. The duplication in the 42 aa repeat variant is placed within the first helix of the repeat, suggesting a weakened interaction with the preceding repeat. In contrast, the aberrant repeat with 40 aa has the duplication in the second helix, suggesting a compromised interaction with the following repeat. Based on these observations, we wondered whether the presence of both duplications in a single repeat would weaken the interactions with both adjacent repeats and result in a repeat that constantly leaves the repeat array. We placed such a synthetic 48 aa aberrant repeat at position 7 (HD) within a TALE with 17.5 repeats and tested the TALE for its activity on an optimal and a −1 frameshift box (Supplementary Figure S6). Indeed, the TALE containing the synthetic 48 aa variant showed only activity on a −1 frameshift box, indicating a strong bias of this repeat variant to loop out of the repeat array (Supplementary Figure S6). This supports a model in which the alteration in an aberrant repeat specifically affects the inter-repeat interactions with either one of the adjacent repeats.

### All out: two aberrant repeats only function independently at a distance

**Figure 3.**
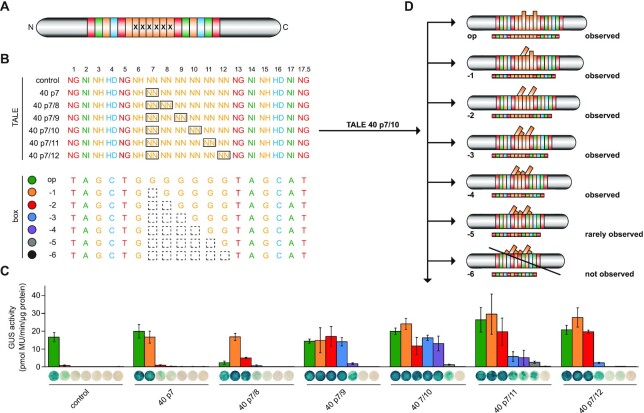
Two distant aberrant repeats can function independently. (**A**) TALE set-up. Altered repeats are indicated by x. (**B**) RVDs of constructed TALEs. Aberrant repeats are boxed. TALE boxes were optimal (op) or frameshift variants with one to six nucleotides deleted (−1 to −6; dashed squares). (**C**) GUS assay of TALEs on boxes shown in panel (B). Error bars represent standard deviation (*n* = 3). Colours refer to the boxes in panel (B). (**D**) Possible binding behaviour of the TALE with aberrant 40 aa repeats at positions 7 and 10 (longer rectangles). Binding likely occurs by looping out none (op), one (−1) or both (−2) aberrant repeats or by looping out both aberrant repeats together with several normal repeats in between (−3 and −4).

The flexible looping-out behaviour of individual aberrant repeats raises the question how the distance between two aberrant repeats influences their behaviour and the overall activity of a TALE. For these experiments, we used the same TALE set-up with 17.5 repeats shown in Figure 2, and placed a 40 aa aberrant repeat at position 7 and another one at position 8, 9, 10, 11 or 12 (Figure 3A). The TALEs were tested together with a control TALE without an aberrant repeat on the series of target boxes with one to six nucleotides deleted (Figure 3B).

All tested TALEs were functional, but differences in their behaviour were observed. Two aberrant repeats placed in direct vicinity preferentially looped out one or both aberrant repeats (Figure 3C). If one or two standard repeats were placed between two aberrant repeats, the respective TALEs were highly active on a wide range of boxes, indicating that both aberrant repeats can stay in (op), one of them can loop out separately (−1) or both of them can loop out simultaneously (−2) (Figure 3C and D). Strikingly, high activity was also observed on the −3 and −4 boxes, which can only be explained by a TALE conformation with three or four repeats not partaking in DNA binding. We hypothesize that two aberrant repeats drag out one or more standard repeats between them while they themselves leave the repeat array, resulting in an extraordinary flexibility of the TALE (Figure 3D). This shows that under specific circumstances also a standard 34 aa repeat can leave the repeat array and not partake in DNA binding. The likelihood to loop out more than two repeats dropped drastically, if three or more standard repeats were placed between the two aberrant repeats (Figure 3C). For two aberrant repeats to show an independent flexible looping-out behaviour, it is therefore advisable to position them with at least three, better four normal repeats in between. We further analysed whether a combination of 42 and 40 aa aberrant repeats together would enhance the cooperative excision of aberrant repeats and normal ones sandwiched between them by weakening the interaction with both outward neighbouring repeats. This was not the case, although the preferences of the TALEs for specific target boxes changed slightly in comparison to ones with two 40 aa repeats (Supplementary Figure S7). This demonstrates that the nature of the two aberrant repeats influences the overall binding behaviour of the respective TALEs.

### Synthetic networks: TALEs for multi-readout expression of logic genetic gates

The independent flexibility of multiple aberrant repeats within one TALE prompted us to test the combinatorial possibilities as synthetic biology transcriptional switches. A series of TALEs with zero to two 40 aa aberrant repeats and an overlapping specificity was designed to match different combinations of highly related target sequences. The TALEs contained 17.5 repeats of alternating specificity and a single aberrant repeat at either position 4 or 12, or two aberrant repeats at positions 4 and 12. In addition, the repeat at position 4 or 12 was omitted (Supplementary Figure S8A–C). We tested the TALEs on a set of boxes lacking the base at either position 4 or 12, or both of them (Supplementary Figure S8A–C). The TALEs showed activity only on their optimal box or on boxes where the nucleotide deletions were placed corresponding to the positions of the aberrant repeats present in the respective TALE (Supplementary Figure S8D). This allowed activation of individual boxes, as well as combinations of two or four boxes. With careful fine-tuning and keeping in mind the requirement for a reasonable number of strong RVDs, it is now possible to address a set of different boxes individually and in multiple possible combinations. By using such a set of TALEs as master switches and inserting the different target boxes as control elements into target promoters, this set-up can be used as a powerful tool for the regulation of genetic circuits (e.g. artificial biosynthetic pathways) with multi-readout expression (Supplementary Figure S8E).

### Natural flexibility: members of class TalBK contain two aberrant repeats and bind to multiple target sequences

**Figure 4.**
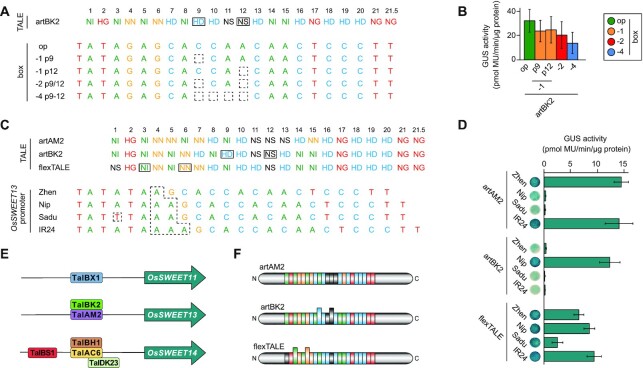
TalBK2 binds within the *OsSWEET13* promoter. (**A**) RVDs of artBK2. Aberrant repeats are boxed (both 36 aa v1). TALE boxes were optimal (op) or frameshift variants with various deletions at positions 9–12 (−1 p9, −1 p12, −2 p9/12 and −4 p9–12). (**B**) GUS assay of constructs in panel (A). Error bars represent standard deviation (*n* = 3). (**C**) RVD composition of artAM2, artBK2 and a TALE designed to address all four shown *OsSWEET13* promoter variants (flexTALE). Aberrant repeats are boxed (36 aa v1). The 1000-bp fragments of the *OsSWEET13* promoter from different rice cultivars (Zhenshan, Zhen; Nipponbare, Nip; Sadu Cho, Sadu; and IR24) were amplified and fused in front of the GUS reporter gene. Only the TALE target region is shown. Differences between cultivars are indicated by dashed boxes. (**D**) GUS assay of constructs shown in panel (C). Error bars represent standard deviation (*n* = 3). (**E**) Representative examples from *X. oryzae* TALE classes targeting *OsSWEET* genes. (**F**) Schematic of constructed TALEs. N- and C-terminal regions are derived from Hax3.

Only two examples of natural TALEs carrying multiple aberrant repeats exist, classes TalHT and TalBK (Supplementary Figure S1). Class TalHT is a truncTALE/iTALE and unable to bind DNA. Class TalBK members were shown to induce the well-known susceptibility gene *OsSWEET13* (19,46). All members of class TalBK share the aberrant repeat variant 36 aa v1 at positions 9 and 12 with the RVDs HD and NS, respectively (Supplementary Figure S9A). The general RVD set-up of these TALEs is extremely similar; however, TalBK1 and TalBK15 are six repeats shorter than the other members, while TalBK1, TalBK3, TalBK4, TalBK11, TalBK12 and TalBK14 contain one different RVD at position 8 (HD instead of NI) (Supplementary Figure S9A). To test the DNA-binding specificity of class TalBK, we constructed three artificial TALEs using the RVDs of TalBK1, TalBK2 and TalBK15, named artBK1, artBK2 and artBK15, respectively (Figure 4A; Supplementary Figure S9B–D). An optimal target box was generated based on the RVDs of TalBK2. Additionally, four different frameshift derivatives of this box were generated, carrying a one-nucleotide deletion at position 9 or 12, a two-nucleotide deletion at positions 9 and 12, or a four-nucleotide deletion from positions 9 to 12. GUS reporter studies *in planta* showed that TALE artBK2 is highly active on the optimal box, as well as on frameshift boxes with nucleotide deletions at either one or both positions corresponding to the aberrant repeats (Figure 4A and B). Furthermore, this TALE showed activity on the box with the four-nucleotide deletion, a box that can only be addressed by looping out the two aberrant repeats together with the two standard repeats between them (Figure 4A and B). ArtBK15, on the other hand, showed only low activity, while artBK1 displayed no activity at all (Supplementary Figure S9E). The lower activity of artBK15 in comparison to artBK2 is likely due to the reduced number of repeats, which results in a lower DNA-binding affinity, while artBK1 is further impaired by a mismatch at position 8 in our set-up. In nature (*Xoo* KACC10331), TalBK1 has an additional frameshift mutation in its C-terminal region, rendering it incapable of gene activation and possibly a pseudogene (Supplementary Figure S9B). We cannot conclude whether TalBK3, TalBK4, TalBK11, TalBK12 and TalBK14 would be able to induce gene activity on the optimal box. While they carry the same RVD change that renders artBK1 unable to induce the optimal box, they are six repeats longer, and thus should be less impaired by a single mismatch than their shorter class member.

### An arms race: TalBK and TalAM activate different *OsSWEET13* promoter variants

We used AnnoTALE v1.4.1 (42,45) to screen the promoterome of the rice cultivar Nipponbare for potential target sites of TalBK2. When we manually removed either one of the two aberrant repeats from the TALE DNA sequence to simulate its loop out, *OsSWEET13* ranked at the top position with a perfectly matching target site (Supplementary Figure S10), which is consistent with previous reports (19,46). The same position in the *OsSWEET13* promoter is also targeted by TalAM2 (PthXo2; Figure 4E) (6,14), which does not contain any aberrant repeat, and it coincides with known INDEL variations in the *OsSWEET13* promoter (6,14). Such promoter variations function as natural resistances by prohibiting binding of TALEs and thereby blocking activation of key virulence genes (6,17,18). We aimed to understand the interaction of the different *OsSWEET13* promoter variants and *Xoo* TALEs in the context of resistance. The *OsSWEET13* promoter of several *indica* and *japonica* rice cultivars was sequenced and one new variant from cultivar Sadu Cho was identified in addition to the known ones from Nipponbare, Zhenshan and IR24. These promoters differ by a number of adenosines in a region that corresponds to the first quarter of the TalBK target site (Figure 4C). The target site in Sadu Cho resembles the one from the cv. Nipponbare, but it carries an additional A-to-T exchange at the position of the first repeat of TalBK. To test how well these promoters can be induced by the *Xoo* TALEs, 1-kb promoter fragments were cloned in front of the GUS reporter gene and tested in combination with artificially assembled versions (artBK1/2/15, artAM2) of the natural TALEs TalBK and TalAM2 in *N. benthamiana*. ArtBK2 was only able to activate the promoter from Nipponbare, whereas none of the other variants was recognized (Figure 4D). ArtBK1, on the other hand, showed no activity at all, while artBK15 induced the promoter fragment from Nipponbare, however, to a lower extend than artBK2 (Supplementary Figure S9F–H). ArtAM2 induced activity at the *OsSWEET13* promoter variants from cultivars Zhenshan and IR24, but not Nipponbare or Sadu Cho (Figure 4C and D). The binding possibilities and resulting mismatches of the TALEs to the different promoter sequences are shown in Supplementary Figures S11–S14. Our results confirm *OsSWEET13* from the cv. Nipponbare as natural target of TalBK2. They also indicate that the one additional mismatch at the critical position 1 in Sadu Cho is sufficient to block artBK2 recognition (Supplementary Figure S11). To demonstrate the potential of a flexible TALE, we designed a TALE to recognize all four promoter varieties (termed flexTALE). For this, we placed two aberrant repeats at positions 3 and 6, and combined those with an unspecific NS RVD at position 1 (Figure 4C and D; Supplementary Figure S14B). This flexTALE was active on all promoter variants (Figure 4D), demonstrating that TALEs containing aberrant repeats can recognize multiple allelic sequences.

To analyse the activity of TalBK2 in a more natural context, we expressed it in *Xoo* strain BAI3Δ*talC*. This strain is an avirulent derivative of the highly virulent *Xoo* strain BAI3 that lacks the major virulence factor TalC (TalBS1). Its pathogenicity can be restored by the introduction of an *OsSWEET*-inducing TALE (11,12). The TalBK2-expressing variant of this strain was inoculated into rice plants from the four rice cultivars Zhenshan, Nipponbare, Sadu Cho and IR24. Disease symptoms developed only in plants from the cv. Nipponbare, while a TalAM2-expressing BAI3Δ*talC* strain caused symptom formation on plants from the cv. Zhenshan and IR24, thus mirroring the results previously obtained in our GUS assay (Supplementary Figure S15). At the same time, the avirulent strain BAI3Δ*talC* did not result in any disease development, while the BAI3 wild-type strain, due to its TalC-dependent induction of *OsSWEET14*, caused disease phenotypes on all four cultivars.

Taken together, our results support the finding that *OsSWEET13* is the natural target of some if not all TalBK members (19,46). The binding of these TALEs to the promoter is most likely achieved by looping out either one of the two aberrant repeats since only these two confirmations result in a situation where the TALE encounters a perfect matching binding sequence in the *OsSWEET13* promoter from the rice cv. Nipponbare.

### A fragile balance: how a TALE repeat becomes aberrant

We observed that not all natural occurring aberrant repeats can confer frameshift binding. This indicates that the position of the modification within the repeat and its amino acid composition control the impact of aberrant repeats on the interaction of TALEs with DNA. To investigate this further, we first focused on 36 aa repeat variants. The natural 36 aa v1 repeat (duplicated positions 27–28) loops out facultatively and allows standard as well as frameshift binding, whereas the 36 aa v2 repeat (duplicated positions 32–33) is fixed within the array in a normal recognition mode (Figure 1). By introducing a 2 aa duplication at several other positions of a TALE repeat, we generated four additional artificial 36 aa repeat variants (Supplementary Figure S16). The aberrant repeats (RVD NN) were placed at position 8 of a TALE with 16.5 repeats and the resulting TALEs were tested for their ability to bind to an optimal or a frameshift box (Supplementary Figure S16). Two variants (art1, art4) were fully flexible aberrant repeats, showing similar behaviour as the natural variant 36 aa v1. The two other variants (art2, art3), however, rendered the TALE completely inactive on any of the target sequences. In these variants, the duplication was inserted relatively close to the RVD loop. This demonstrates once more that even a single repeat with an unfavourable amino acid composition is able to generally block the activity of a TALE.

### Linker scanning: amino acid composition and position of repeat insertions control their behaviour

To study how the amino acid composition of an aberrant repeat influences its behaviour, we created variants of the two natural 40 and 42 aa aberrant repeats. We replaced each of the duplicated parts present in the natural variants by a stretch of serines and glycines (SG) of identical length (Supplementary Figure S17). Chains of serines and glycines are often used as unstructured linkers to connect protein domains. These artificial aberrant repeats were placed at position 8 in a TALE with 16.5 repeats and tested for activity on an optimal and a −1 frameshift box. The two TALEs with artificial 40 aa variants show opposite behaviour. While the variant with the first half of the duplication replaced strongly prefers the −1 frameshift box, the variant with the second half replaced prefers the optimal box (Supplementary Figure S17). If the SG linker replaced the first half of the duplication in the variant with 42 aa, the TALE showed activity on both target boxes. In contrast, if the second half was replaced, no activity at all was detected (Supplementary Figure S17). These striking differences between the natural repeats and artificial variants show that the amino acid composition of an aberrant repeat controls its impact on TALE activity.

**Figure 5.**
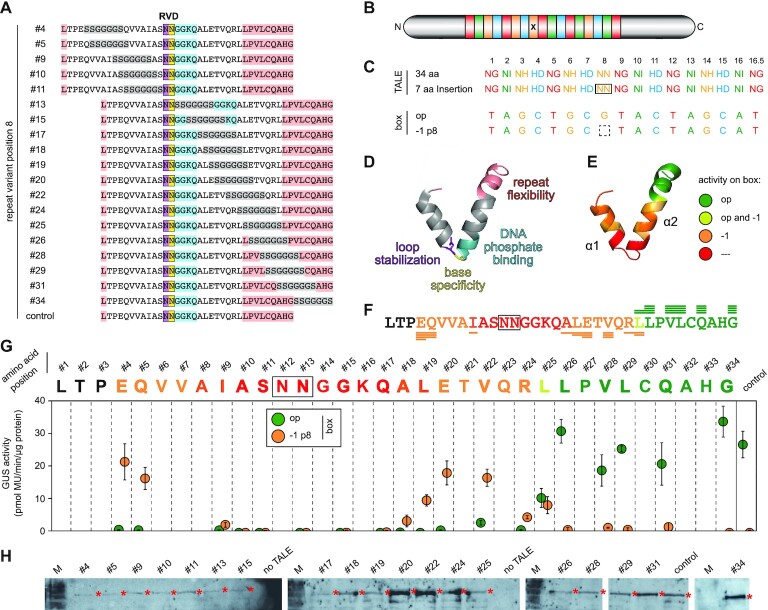
Tolerance of a TALE repeat for insertions. (**A**) Constructed repeat variants. A 7 aa serine–glycine linker (SSGGGGS) was placed at different positions (#) throughout a TALE repeat. RVDs, boxed; inserted amino acids, grey. Functional motifs are coloured according to panel (D). (**B**) TALE set-up. The altered repeat is indicated (x). (**C**) RVDs of constructed TALEs with the repeat at position 8 containing the different linker insertions. TALEs were tested on an optimal box (op) and a −1 frameshift derivative (−1 p8). (**D**) Functional motifs found within a TALE repeat according to Deng *et al.* (66). (**E**, **F**) Tolerance of a TALE to the insertion of 7 aa serine–glycine linkers in a single repeat based on the data in panel (G). Colour code: activity at optimal box (no looping out), green; activity at frameshift sequences (looping out), orange; flexible binding, light green; loss of TALE activity, red; no data, black. Number of lines below the amino acids correspond to relative activity in panel (G). RVDs, boxed. (**G**) GUS assay of TALEs with repeat variants on boxes shown in panel (C). Error bars represent standard deviation (*n* = 3). (**H**) Expression of the TALEs *in planta* was confirmed after immunoblotting using an anti-GFP antibody. Asterisks indicate expected sizes.

Next, we wanted to see how the position of an alteration within an aberrant repeat affects its behaviour. We therefore introduced a 7 aa linker sequence (SSGGGGS) at different positions throughout a TALE repeat (Figure 5A) and inserted those repeats into a TALE at position 7 (Figure 5B and C). We then tested those TALEs on an optimal box (op) and a −1 nucleotide frameshift box with a deletion at position 7 (−1 p7). We noticed that the positions close to the end of a TALE repeat (positions 26–34) tolerate the insertion of the 7 aa linker without any change in the binding behaviour, leading to activity only on the optimal box (Figure 5E–G). Insertions at positions 4–5 within the first α-helix (α1) or positions 19–24 within the second α-helix (α2) of the repeat, on the other hand, resulted in a compulsory loop out of the repeat (Figure 5E–G). The only repeat variant that conferred flexible binding to both the optimal and the frameshift box was the one with an insertion at position 25. If the linker was placed in the region around the RVD loop (positions 9–18), it resulted in a complete or near-complete loss of TALE function (Figure 5E–G). Western blot analysis demonstrated that this loss of function is not attributed to loss of protein expression or stability (Figure 5H). A second experiment confirmed this pattern and showed that the insertion of even a single amino acid (serine, S) into or around the RVD loop of a TALE repeat is sufficient to impair the overall activity of a TALE drastically (Supplementary Figure S18). These results clearly show that amino acid insertions into the regular structure of TALE repeats have a differential impact on their behaviour. Disturbances of the regions flanking the RVD loop destroy overall function of the TALE, whereas insertions into either one of the helical regions disturb repeat-to-repeat interactions and favour looping out of the aberrant repeat. Finally, insertions near the loop region between repeats (e.g. the end of a repeat) are tolerated without impact on the DNA-binding behaviour of the TALE.

### Functional fusions: TALEs tolerate insertions of large domains between repeats

**Figure 6.**
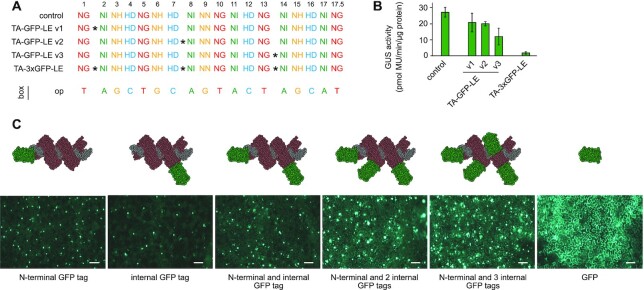
Insertion of foreign domains into TALE repeats. (**A**) RVDs of constructed TALEs. GFP (*) was inserted into the TALE repeat array at three different positions (TA–GFP–LE v1–v3) or at all three positions simultaneously (TA–3×GFP–LE). (**B**) GUS assay of TALEs on boxes shown in panel (A). Error bars represent standard deviation (*n* = 3). (**C**) Confocal microscopy images of GFP or GFP-tagged TALEs. Size bar is 100 μm. The set-up of the different constructs is visualized by cartoons.

Because the insertion of a 7 aa linker close to the end of a repeat was well tolerated, we focused on placing inserts between two repeats. First, we inserted two serine/glycine linkers of 7 and 18 aa, respectively, between repeats 7 and 8. The resulting TALEs showed activity only on the optimal box (Supplementary Figure S19A–C). Next, we tested whether it is possible to insert larger protein domains between two repeats. We combined a 238 aa long GFP protein with a 7 aa SG linker on each end and placed it between the repeats at positions 7 and 8 (Figure 6). To determine whether the TA–GFP–LE fusion protein is still able to bind to a specific target sequence, a GUS assay on an optimal box (op), on a −1 frameshift box (−1 p8) and on a +1 frameshift box was performed. The TA–GFP–LE resulted in a comparable activity to the control TALE with standard repeats on the optimal box (Supplementary Figure S19D-F). Both had no activity on a +1 box, indicating that the large size of the GFP protein does not impose a gap upon the standard repeat-to-repeat assembly of the regular TALE repeat structure, but likely loops out of the array, similar to an aberrant repeat. We also observed a low but reproducible activity for a TALE carrying a GFP insertion in the repeat array on a −1 frameshift box, indicating that sometimes one of the standard repeats adjacent to the GFP insertion loops out as well (Supplementary Figure S19D–F). The GFP insertion could be placed at different positions throughout the repeat array (position 2, 8 or 14) with similar results (Figure 6). However, a TALE with two or more GFPs inserted at different positions showed strongly reduced activity, which suggests that the protein cannot interact properly with target DNA sequences (Figure 6). To clarify whether all parts of the TA–GFP–LE are bound to the DNA, we designed three different mismatch boxes, harbouring three mismatches at position #3–5, #10–12 or #14–16 (Supplementary Figure S19G). This analysis confirmed that indeed all parts of the TA–GFP–LE are bound to DNA (Supplementary Figure S19H). Laser scanning microscopy showed that the GFP domain placed within the repeat array is properly folded and fluorescing (Figure 6C). These data show that it is possible to insert large functional domains in between individual repeats of a TALE without interfering with its specific DNA recognition *in vivo*. This opens exciting avenues to expand the TALE scaffold in the third dimension for new biotechnology applications.

## DISCUSSION

TALEs are unique DNA-binding proteins with a simple and strictly regular DNA-binding pattern. Each repeat in a TALE is recognizing exactly one DNA base in a consecutive fashion (23,24,27,28). This modular structure enables the creation of TALEs with novel DNA-binding specificities by duplication, deletion and recombination of repeats (47). Such adaptability is typical for virulence factors since it ensures the steady emergence of novel weapons in the evolutionary arms race between host and pathogen (48–50). Still, the strict regularity of TALEs renders them particularly susceptible to frameshift mutations in their target DNA sequence, which inevitably result in multiple repeats being out of sync with their target DNA bases. Accordingly, natural mutation of TALE target sequences in host promoters, which prohibit TALE binding, can be readily found (6–8,13–16,47). Aberrant repeats can be seen as the next step in this arms race, allowing for the parallel recognition of different allelic promoter variants.

Aberrant repeats seem to exist in all Asian, but not in African, *X. oryzae*. This suggests that the selection of rice cultivars during the particularly long domestication of rice in Asia (51) has accelerated their emergence in the arms race between plants and pathogens. Still, the generation of a functional aberrant repeat is likely a rare event since we found that aberrant repeats of the same type are typically located in TALEs of one or several related classes and mostly occur in the same genomic TALE clusters. Accordingly, the emergence of a TALE with multiple aberrant repeats in nature is even rarer and has occurred only once. However, while this TALE and its class members (TalBK) resemble the known virulence factor TalAM2 (PthXo2) in overall RVD composition and virulence target, they recognize the rice cultivar Nipponbare allele of the *OsSWEET13* promoter, which has so far been classified as a resistant allele (6,14). *OsSWEET13* is well known, as it is one of five clade III *SWEET* genes in rice that can support vascular spreading of *Xoo* (12). Three of these *SWEET* genes are targeted at different positions in their promoters by seven different TALEs originating from different *Xoo* strains, which emphasizes the importance of sugar efflux into the xylem for *Xoo* infection (12,48). In fact, many *Xoo* strains contain two different *SWEET*-inducing TALEs. PXO142, for example, carries both TalBH (PthXo3) targeting *OsSWEET14* and TalBK targeting *OsSWEET13*. Such redundancy is powerful since it allows to effectively break resistances that are based on mutation of only one of the two virulence targets. We identified the *SWEET13* allele in rice cultivar Sadu Cho, which is similar in composition to Xa25-3 (19), as another resistant variety that cannot be targeted by any of the known TALEs. This opens the possibility of using this cultivar as a novel source for breeding resistant rice.

In this study, we have scanned published *Xanthomonas* genomes for TALEs with aberrant repeats. Functional analysis revealed that many but not all of these aberrant repeats allow for a flexible frameshift recognition. Some, like the 35 aa variant, the 36 aa variant 2, and the 37 aa variant 1, behave like standard repeats, while the 28 aa variant and 37 aa variant 2 loop out predominantly (Supplementary Figure S20). Aberrant repeats with 28 aa occur only in TALEs that all share N- and C-terminal truncations. These TALEs do not bind to DNA and are unable to activate gene expression—instead they suppress a resistance protein that can recognize TALEs. Accordingly, they were named truncTALEs (41) or iTALEs (40). So far, no specific role has been found for the 28 aa repeat in these TALEs and it is unclear why it evolved.

We explored the capability of TALEs to accommodate more than one aberrant repeat and the implications for DNA binding. In a consecutive array of aberrant repeats, multiple repeats loop out, likely because the following repeats move forward such that all DNA-binding repeats interact with consecutive DNA bases without leaving a gap. This implies that the looped-out repeats are accommodated in such a way that the repeat-to-repeat interactions of the other repeats are not compromised, possibly by forming a folded subdomain outside of the DNA-bound repeat array. There seems to be a limit to this system, though, because five and six aberrant repeats in tandem resulted in a TALE with very low overall activity. This suggests that the subdomain enforces structural constraint if it becomes too large.

When two aberrant repeats are placed into an array of standard repeats, their relative distance determines how they affect DNA binding. If one or two normal repeats are placed in between, the aberrant repeats can apparently loop out independently or together with the normal ones. This shows that the looped-out subdomain can also include standard repeats. Interestingly, if three or four normal repeats are located between two aberrant repeats, each aberrant repeat exhibits a flexible and independent looping-out behaviour. Therefore, if TALEs are designed with multiple aberrant repeats that should loop out independently, those aberrant repeats have to be positioned at least four repeats apart. Using this design, we were able to build synthetic gene switches based on TALEs with one or two aberrant repeats to target different combinations of related target DNA boxes with very high specificity. Such boxes could be used in combination with different hierarchical levels of TALE master switches to control expression of individual, overlapping or alternative combinations of genes in synthetic biology applications.

Our data imply that many aberrant repeats weaken the repeat-to-repeat interface, which causes them to loop out such that their position in the array is replaced by the following standard repeat. Most of the natural aberrant repeats contain short duplications in either one of the two alpha-helical regions of the repeat. This appears to be necessary for their flexible behaviour since the flexibility is lost if one of the duplicated regions is replaced by a linker sequence. This observation can be explained by the fact that the alpha-helical regions are responsible for inter-repeat interactions within the repeat structure (27,28). The first helix forms hydrophobic interactions with the second helix of the preceding repeat and the second helix forms hydrophobic interactions with the first helix of the following repeat. It is conceivable that these interfaces allow for both the flexibility and the cohesion of the superelastic spring-like TALE structure (52).

Two other types of repetitive proteins facilitate binding to nucleotides in a one-repeat-to-one-nucleotide manner, PPRs (pentatricopeptide repeats) and PUFs (Pumilio and FBF); however, their binding specificity is governed by two and three amino acids, respectively, and they recognize single-stranded RNA (Supplementary Figure S21) (53,54). Interestingly, repeats of aberrant length exist also in PRRs and PUFs, but typically they do not show a different binding mode (55–57). However, the two PUFs Puf4 and FBF contain an insertion of 16 aa and recognize a target sequence with an additional nucleotide. The aberrant repeat in those proteins apparently causes the additional RNA nucleotide to loop out, which is the opposite of the mechanism observed by aberrant repeats in TALEs (58,59).

Strikingly, even a single repeat with an insertion close to the RVD loop renders the complete TALE nonfunctional. This is the case even though the amino acids lysine and glutamine at positions 16 and 17 (K16, Q17) are present, which interact with the DNA backbone (27,28). We postulate that a repeat with insertions close to the RVD loop is sterically blocked and cannot condense onto the individual DNA base upon switch from the loose conformation of the scanning mode to the tight conformation of the binding mode (29,30). This tight conformation is characterized not only by RVD–base interactions, but also by precise positioning of K16 and Q17 to the negatively charged DNA backbone, effectively locking a repeat into place (27,28,60). Disturbances in this region might block DNA contact as well as proper positioning of the following repeat helices. TALE proteins are significantly elongated in DNA-free mode in comparison to the bound state [60 Å versus 35 Å for 11.5 repeats (27)]. Initial DNA contact and nonspecific DNA binding is facilitated by the N-terminal domain of a TALE (61) and the first few repeats close to the N-terminal domain are more important for binding than later ones (62–64). If two TALE binding sites overlap, the TALE with a blocked N-terminal region is readily displaced (65). It is intuitive to envisage that condensation of the TALE to the target DNA bases starts at the first repeats and progresses to the C-terminal ones in a zipper-like fashion. Accordingly, if one repeat cannot condense, it might block the downstream repeats such that they can also not properly align to the DNA bases. Such misaligned TALEs will not stay for a prolonged time at this locus, but switch again into search mode along the DNA and result in no activation of downstream genes.

We identified the hinge region between TALE repeats, which forms a repeat-to-repeat loop (27,28), as a place where amino acid insertions are readily tolerated (Figure 5). This region is reported as crucial for the structural plasticity and flexibility of TALE repeats (66). We could show that it even allows for the insertion of a full-length GFP without losing DNA binding of the TALE. This region protrudes to the external area of the TALE–DNA complex and might therefore spatially allow additional protein domains, similar to the arrangement of looped-out aberrant repeats. However, the N- and C-terminal ends of a folded GFP protein are located in close proximity. Since this might affect how well it is tolerated, it could be a prerequisite required for functionality by helping to avoid steric hindrances. It is nonetheless promising that this particular region can now be used to insert other functional domains to add novel features to DNA-bound TALEs. This might be another way to expand the use of TALEs as molecular Swiss Army knives for genome editing or synthetic biology applications.

## DATA AVAILABILITY

All strains and plasmids are available upon request.

## Supplementary Material

gkac098_Supplemental_File
