# Supplementary material for: Flexible TALEs for an expanded use in gene activation, virulence and scaffold engineering
Source: Nucleic Acids Res. 2022 Feb 12;50(4):2387–400. doi: 10.1093/nar/gkac098 (PMC8887545; doi:10.1093/nar/gkac098)
Supplement: gkac098_Supplemental_File [file gkac098_supplemental_file.pdf]

Supplementary Material

Flexible TALEs for an expanded use in gene activation, virulence, and scaffold engineering

Sebastian Becker[1], Stefanie Mücke[1], Jan Grau[2] and Jens Boch[1,*]

[1] Department of Plant Biotechnology, Institute of Plant Genetics, Leibniz Universität Hannover, 30419 Hannover, Germany
[2] Institute of Computer Science, Martin Luther University Halle-Wittenberg, 06120 Halle (Saale), Germany

* To whom correspondence should be addressed.
Tel: +49-511-762 4082
Fax: +49-511-762 4088
Email: jens.boch@genetik.uni-hannover.de

LIST OF SUPPLEMENTARY MATERIAL

Supplemental Figures 1 - 21
Supplemental Tables 1 - 4

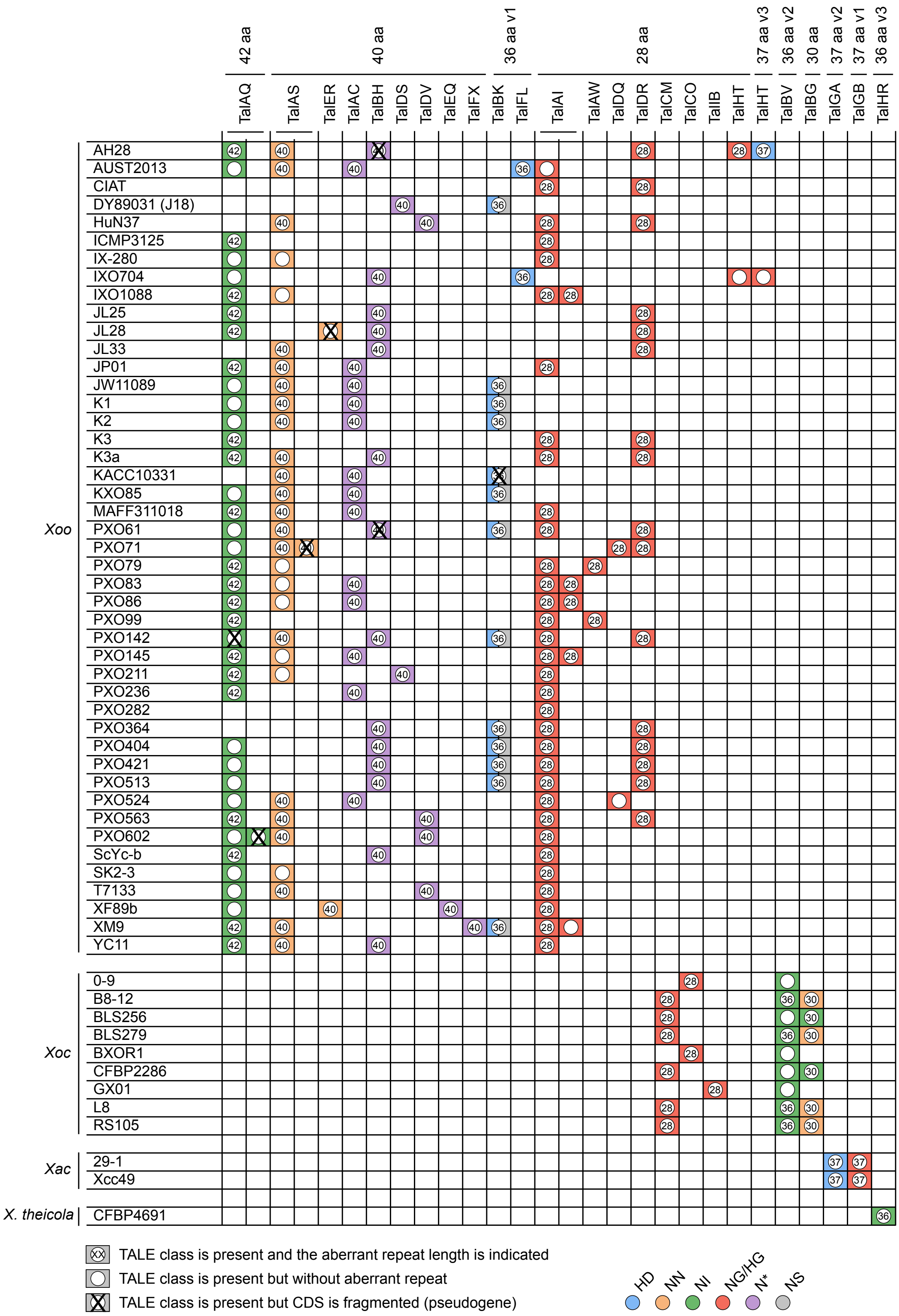

Supplemental Figure 1: Aberrant repeats in *Xathmonas* TALEs. Shown are all TALE classes with at least one member carrying an aberrant repeat. Only completely sequenced *Xanthomonas* strains are included. TALE names according to AnnoTALE. *X. oryzae* pv. *oryzae*, *Xoo*; *X. oryzae* pv. *oryzicola*, *Xoc*; *X. axonopodis* pv. *citri*, *Xac*. Colour indicates RVD of the aberrant repeat. TALEs with the 28 aa aberrant repeat also contain N- and C-terminal truncations that render them unable to bind to DNA (iTALEs/truncTALEs).

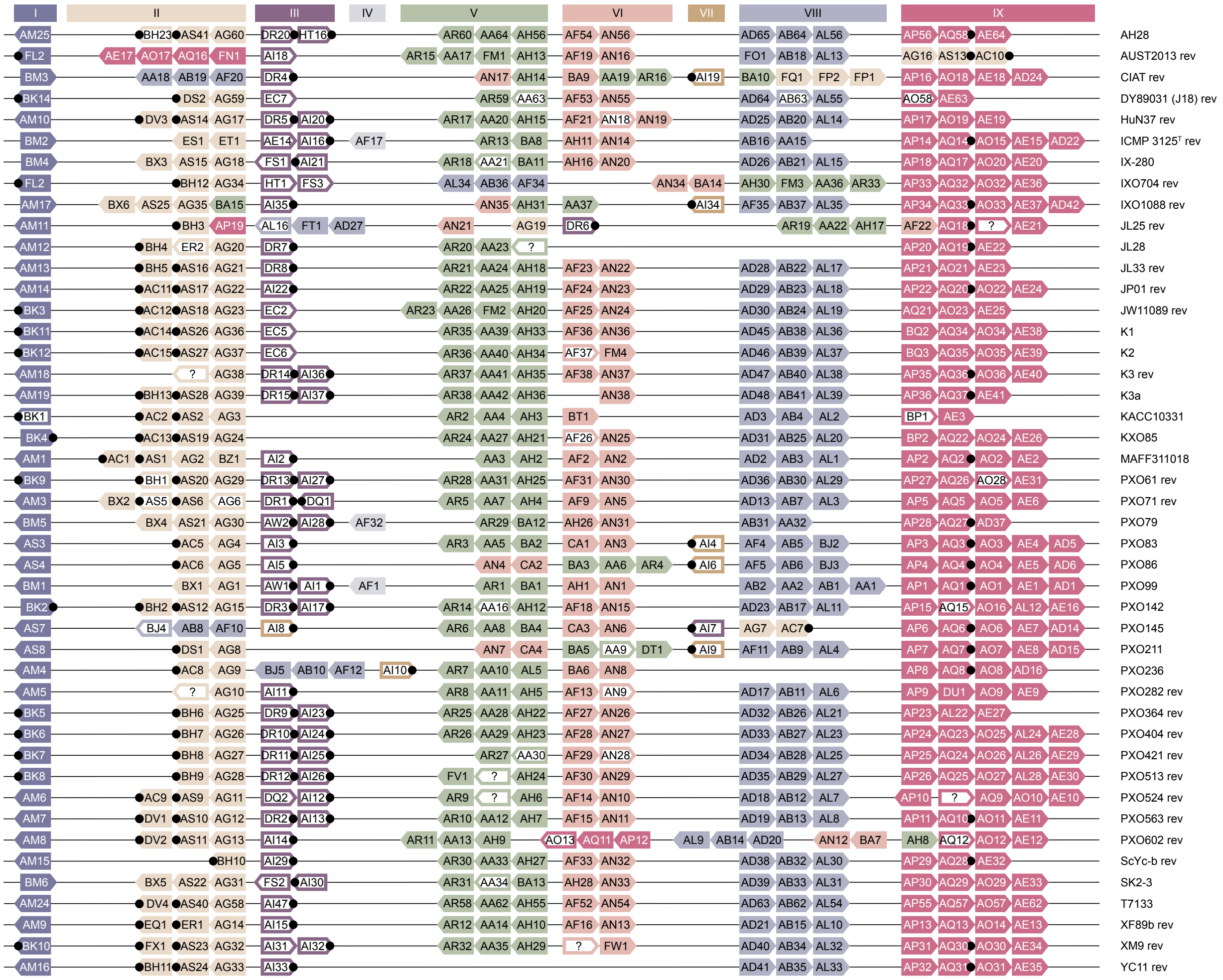

Supplemental Figure 2: Affiliation of TALEs in genomic clusters. Shown are completely sequenced *Xanthomonas oryzae* pv. *oryzae* strains with at least one aberrant repeat. *TALE* genes are depicted as arrows with regard to their orientation in the genome. TALEs are named according to the AnnoTALE classification. Colours differentiate previously established genomic TALE clusters. TALEs carrying an aberrant repeat are indicated by a black dot. Hollow arrows indicate TALEs with incomplete N- and C-terminal regions or frameshifts (pseudogenes or trunc/iTALEs). If AnnoTALE detected a fragmented TALE but was unable to classify it, the gene is labelled with a question mark. Strains shown in reverse orientation are indicated (rev.). This overview includes *Xoo* strains from Asia, Australia and South America since only they were found to carry aberrant repeats.

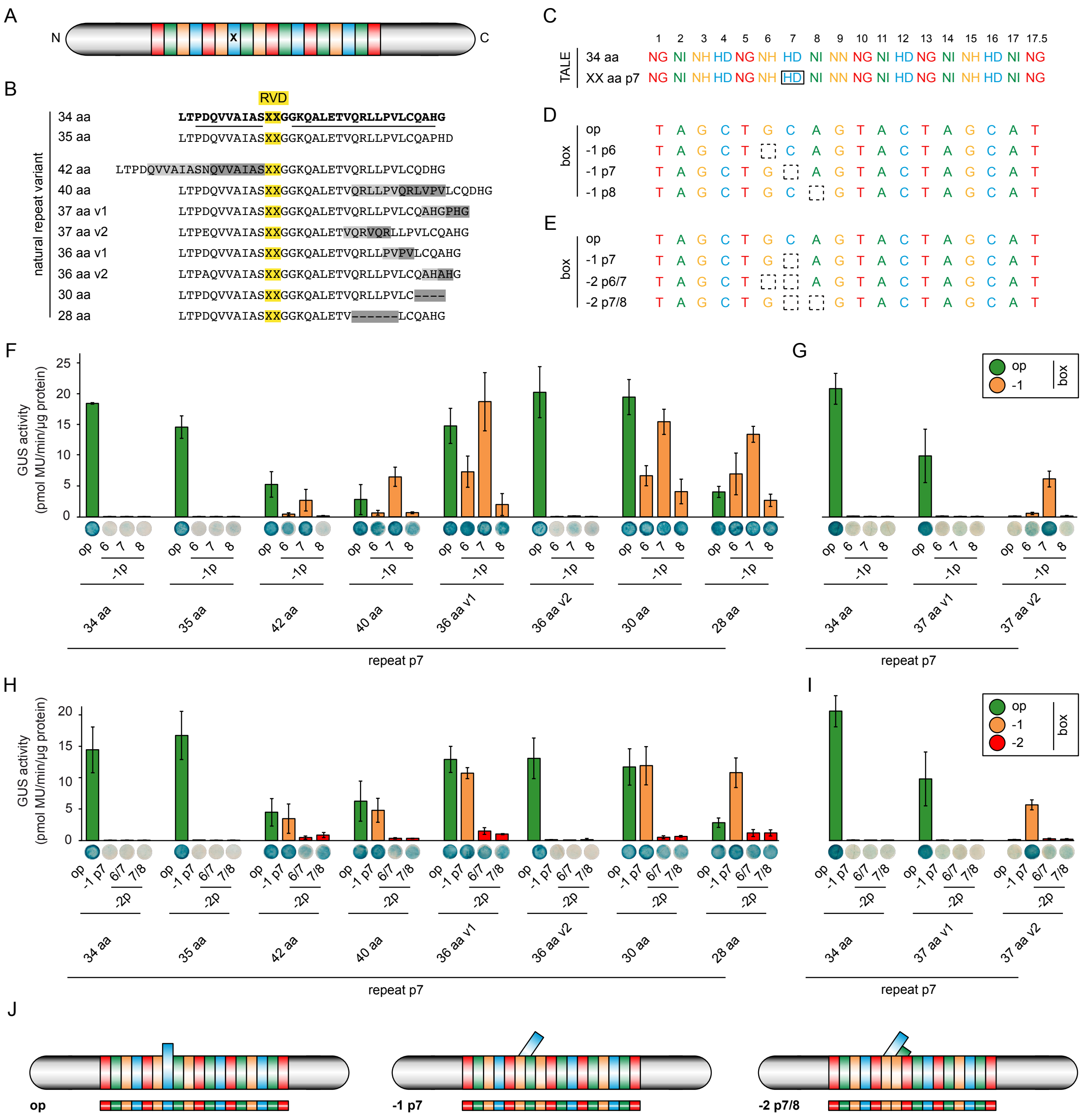

Supplemental Figure 3: TALEs with a functional aberrant repeat prefer target boxes with a -1 nucleotide deletion corresponding to the position of the aberrant repeat. (**A**) TALE setup. The altered repeat is indicated (x). (**B**) Amino acid alignment of the different repeat variants. A standard 34 aa repeat is shown in bold with helix-forming residues underlined. RVDs (yellow), duplicated or deleted aa (grey). (**C**) RVD composition of the artificial TALEs. Position of the aberrant repeat is boxed. (**D**) The TALE boxes were optimal (op), or frameshift variants with one nucleotide deleted at position 6, 7 or 8 (-1 p6, -1 p7 or -1 p8). (**E**) In an alternative setup, the same TALEs were tested on an optimal box (op) and on frameshift variants with either a single nucleotide deletion (-1 p7) or 2 nucleotide-deletions (-2 p6/7 or -2 p7/8). (**F**) and (**G**) GUS assay of TALEs in combination with the target boxes shown in (D). (**H**) and (**I**) GUS assay of TALEs in combination with the target boxes shown in (E). Error bars represent standard deviation (n = 3). (**J**) Schematic view of a TALE with a functional aberrant repeat at position 7 looping out none, one or two repeats if combined with an optimal box, a matching -1 and a -2 frameshift box, respectively. Aberrant repeats (longer rectangles), looped out repeats (tilted rectangles).

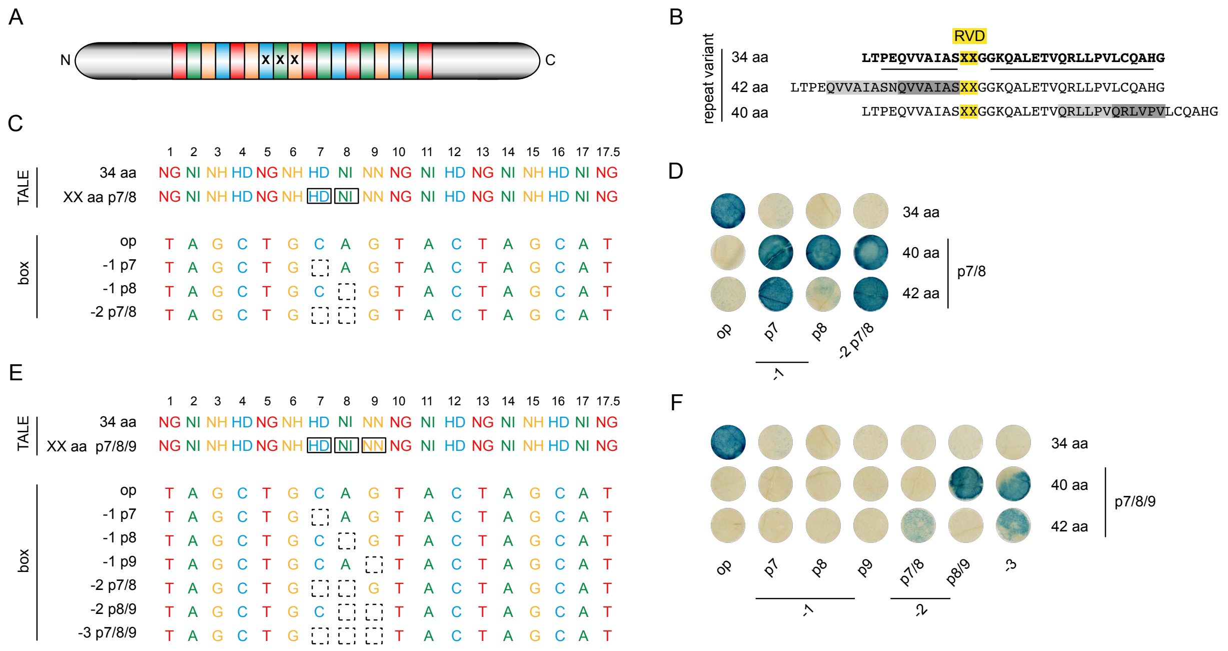

Supplemental Figure 4: Placed in tandem, the 40 and 42 aa aberrant repeats result in TALEs with different target box preferences. (**A**) TALE setup. The altered repeats are indicated (x). (**B**) Amino acid alignment of the different repeat variants. A standard 34 aa repeat (bold), helix-forming residues (underlined), RVDs (yellow) and duplicated aa (grey). (**C**) and (**E**) TALE setup. Positions of the aberrant repeats are boxed. The TALE box was optimal (op), or a frameshift variant with one (-1 p7, -1 p8 or -1 p9), two (-2 p7/8 or -2 p8/9) or three nucleotides (-3 p7/8/9) deleted. (**D**) and (**F**) Results of the qualitative GUS analysis. A representative leaf disc is shown for each combination.

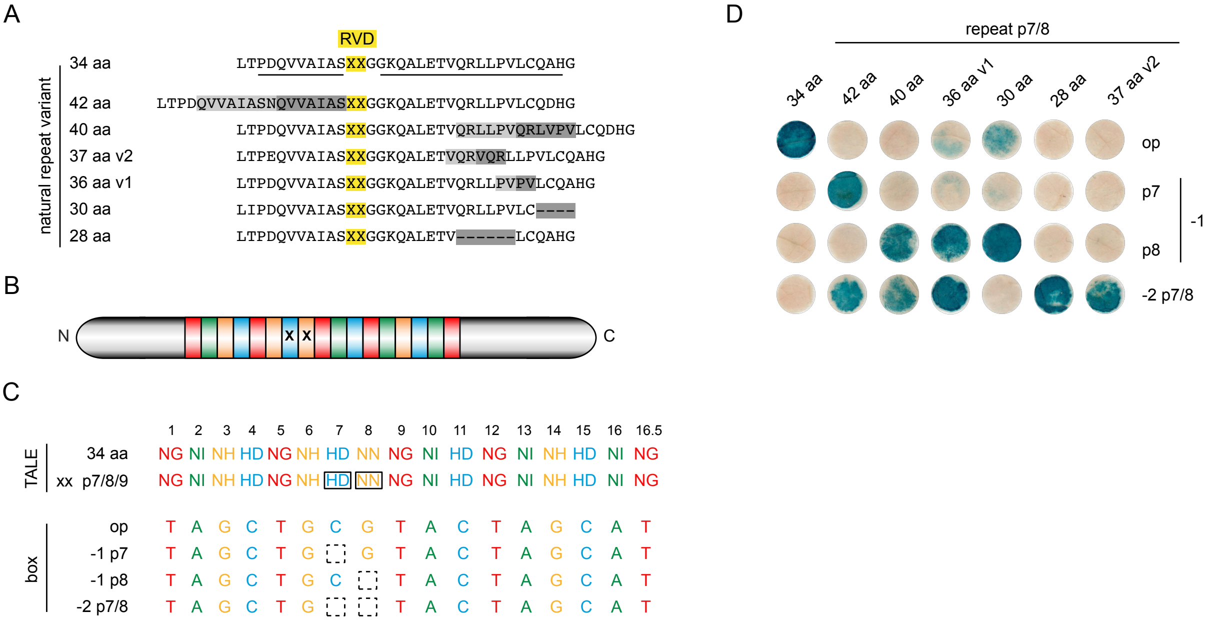

Supplemental Figure 5: TALEs with different aberrant repeats in tandem recognize different target boxes. (**A**) Amino acid alignment of the different repeat variants. A standard 34 aa repeat is shown in bold with helix-forming residues underlined. RVDs (yellow), duplicated or deleted aa (grey). (**B**) TALE setup. The altered repeats are indicated (x). (**C**) RVDs of generated TALEs. Positions of the aberrant repeats are boxed. The TALE box was optimal (op), or a frameshift variant with one (-1 p7 and -1 p8) or two nucleotides (-2 p7/8) deleted. (**D**) Results of the qualitative GUS analysis. A representative leaf disc is shown for each combination.

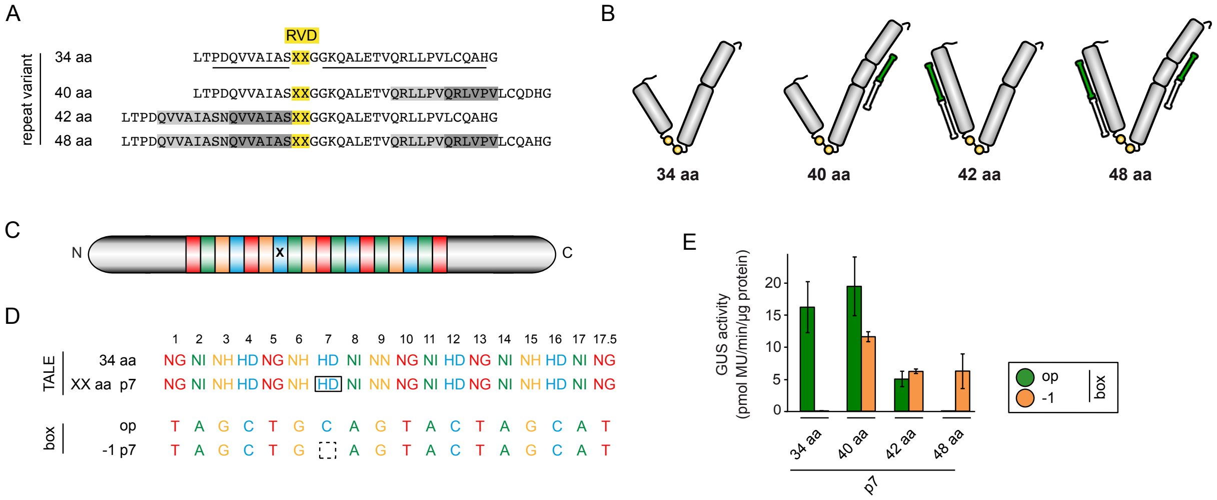

Supplemental Figure 6: An artificial repeat with 48 aa loops out constitutively. (**A**) Amino acid alignment of the different repeat variants. Helix-forming residues (underlined), RVDs (yellow), duplicated aa (grey). The aberrant repeat with 48 aa was generated artificially by combining the duplication from the naturally occurring 40 and 42 aa aberrant repeats. (**B**) Cartoons showing the differences between the aberrant repeat variants. White and green markings indicate duplicated regions. (**C**) TALE setup. The altered repeat is indicated (x). (**D**) RVDs of generated TALEs. Position of the aberrant repeat is boxed. The TALE box was either optimal (op) or a frameshift variant with a one nucleotide deletion at position 7 (-1 p7). (**E**) GUS assay of TALEs on boxes shown in (D). Error bars represent standard deviation (n = 3).

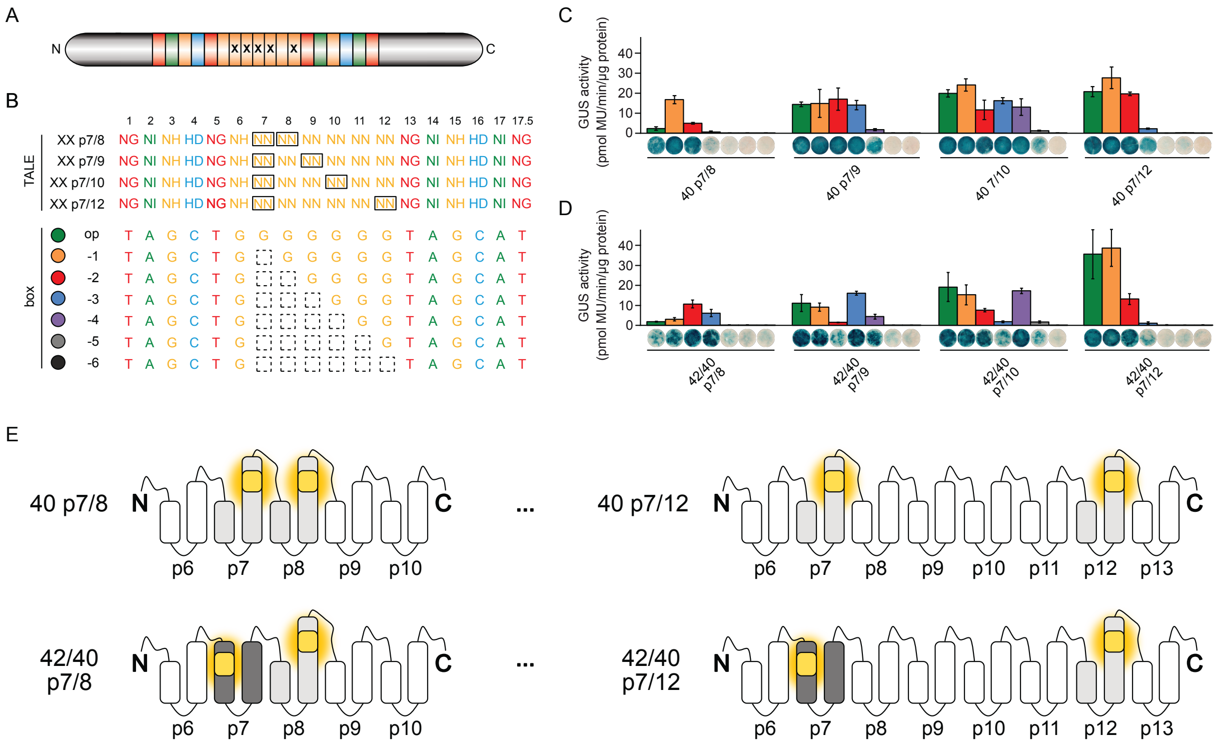

Supplemental Figure 7: The nature of two aberrant repeats influences the loop out behaviour of TALEs. (**A**) TALE setup. Altered repeats are indicated by x. (**B**) RVDs of constructed TALEs. Aberrant repeats are boxed. TALE boxes were optimal (op) or frameshift variants with 1 to 6 nucleotides deleted (-1 to -6; dashed squares). (**C**) and (**D**) GUS assay of TALEs on boxes shown in (B). Error bars represent standard deviation (n = 3). Colours refer to the boxes in (**B**). A representative leaf disc of the qualitative GUS analysis is shown for each combination. (**C**) Both aberrant repeats in each TALE are 40 aa variants. (**D**) The first aberrant repeat in each TALE is a 42 aa variant and the second one a 40 aa variant (duplication in the second helix). (**E**) Cartoon of TALEs used in (**C**) and (**D**). Note that the duplication in 40 aa repeats (light grey) is in the second helix and in 42 aa repeats (dark grey) in the first helix.

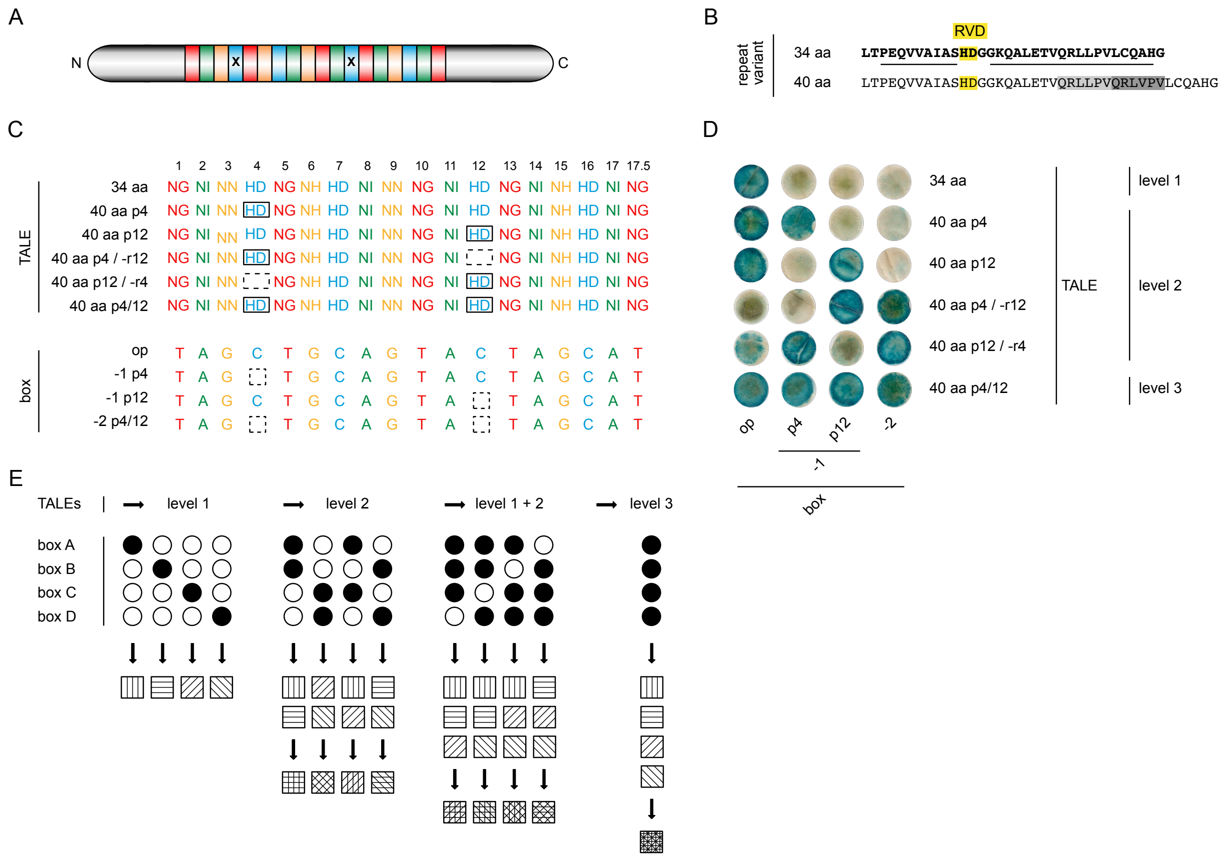

Supplemental Figure 8: TALEs with two properly spaced aberrant repeats remain specific. (**A**) TALE setup. The altered repeats are indicated (x). (**B**) Amino acid alignment of the two utilized repeat variants. RVDs (yellow), duplicated aa (grey). (**C**) RVDs of generated TALEs. Positions containing the 40 aa aberrant repeat are boxed. Omitted repeats are indicated by dashed boxes. The TALE box was optimal (op) or a frameshift derivative containing either a one (-1 p4 or 1- p12) or a two (-2 p4/12) nucleotide deletion. Deleted positions are indicated (dashed boxes). (**D**) Results of the qualitative GUS analysis. A representative leaf disc is shown for each combination. (**E**) Cartoon showing a set of TALE regulators and their different combinatorial possibilities. One, two and four target boxes can be induced using individual level 1, level 2, and level 3 TALEs, respectively. A combination of level 1 and 2 TALEs is required to induce three target boxes,

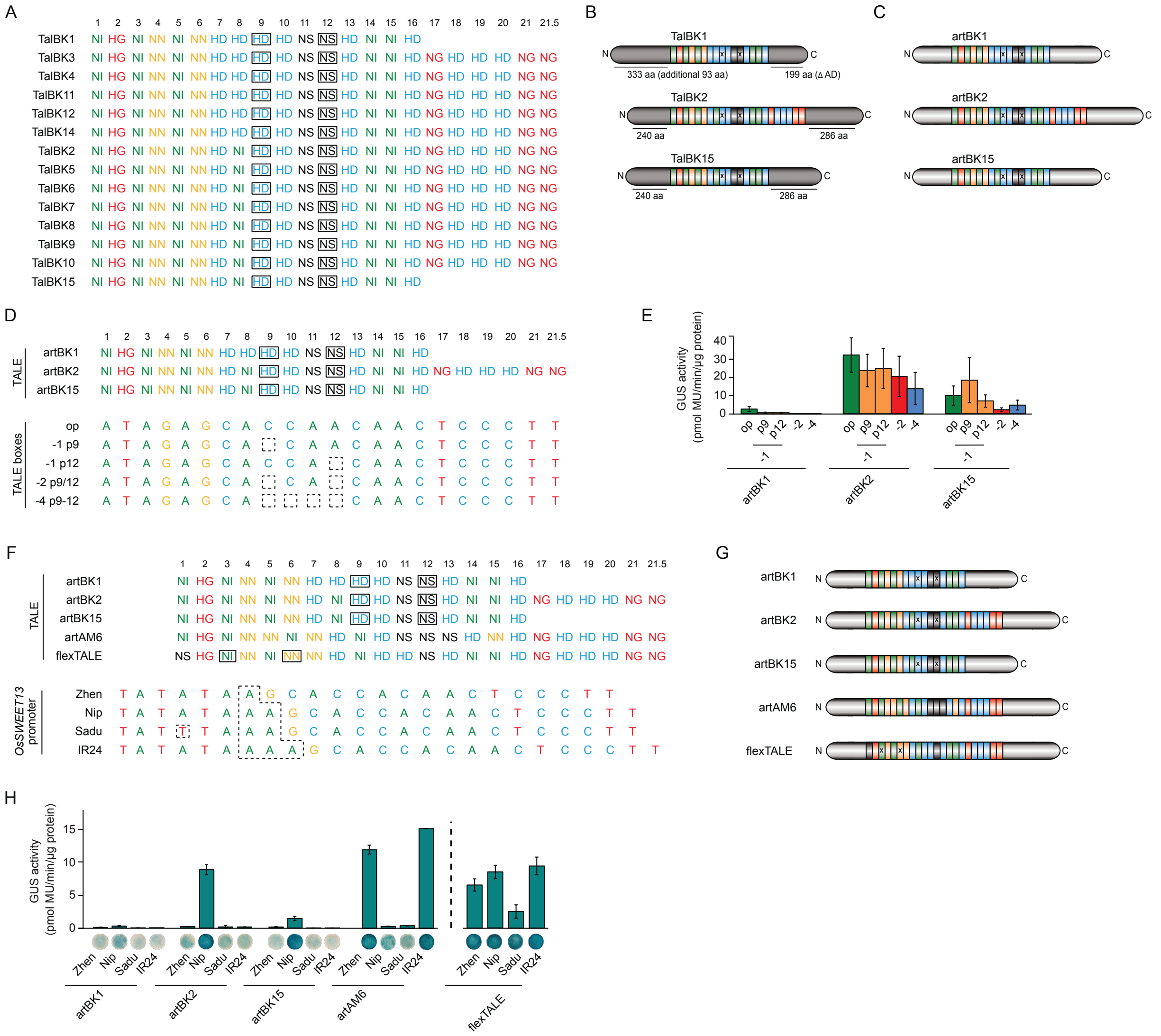

Supplemental Figure 9: Class TalBK members and their ability to bind the *OsSWEET13* promoter. (**A**) Overview of class TalBK. Aberrant repeats are boxed (36 aa v1). Schematic of (**B**) the three natural TALEs TalBK1, TalBK2 and TalBK15 as well as (**C**) their artificially constructed counterparts artBK1, artBK2 and artBK15. Aberrant repeats are indicated (x). N- and C-terminal regions for the constructed TALEs were derived from Hax3. (**D**) RVDs of the constructed TALEs. TALE boxes were optimal (op) or frameshift variants with various deletions at positions 9 to 12 (-1 p9, -1 p12, -2 p9/12 and -4 p9-12). (**E**) GUS assay of TALEs shown in (D). Error bars represent standard deviation (n = 3). (**F**) RVD composition of artBK1, artBK2, artBK15, artAM2 and a TALE designed to address all four shown *OsSWEET13* promoter variants (flexTALE). Aberrant repeats are boxed (36 aa v1). 1000 bp fragments of the *OsSWEET13* promoter from different rice cultivars (Zhenshan, Zhen; Nipponbare, Nip; Sadu Cho, Sadu; IR24) were amplified and fused in front of the β-Glucuronidase (GUS) reporter gene. The TALE-target region is shown. (**G**) Schematic of TALE artBK1, artBK2, artBK15, artAM6 and the flexTALE. Aberrant repeats are indicated (x). (**H**) GUS assay of TALEs on promoters shown in (F). Error bars represent standard deviation (n = 3).

	#	gene	function	position	score	target sequence / mismatches
A TalBK2 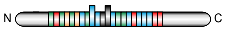 with all repeats binding	1	*Os02g47520.1*	expressed protein	193	-16,328	TATAGAGCATCAACAACACCATA
	2	*Os03g18454.2*	gamma-interferon-inducible lysosomal reductase	308	-16,582	TAAAATGCACCCACACCTCCCCT
	3	*Os06g48350.1*	CPuORF14 - conserved peptide uORF	83	-17,525	TCAATCACACCATCAACTCCCTT
	4	*Os05g05580.1*	expressed protein	311	-17,731	CACAAACCACCACCACCTCCCAT
	5	*Os10g37740.1*	CGMC_GSK.9 -CGMC includes CDA, MAPK, ...	264	-17,887	TCTCCACCACCACCACCTCCCCT
B TalBK2 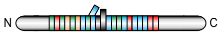 without repeat p9	1	*Os12g29220.1*	nodulin MtN3 family protein (*OsSWEET13*)	267	-7,653	TATAAAGCACCACAACTCCCTT
	2	*Os03g20090.1*	MYB family transcription factor	365	-15,959	TATAAACCCCGACACCTCCCTC
	3	*Os06g24960.1*	retrotransposon protein, putative	215	-16,103	TTCACATCAAAACAACTCCCTT
	4	*Os03g44360.1*	retrotransposon protein, putative	215	-16,103	TTCACATCAAAACAACTCCCTT
	5	*Os11g23244.1*	retrotransposon protein, putative	215	-16,103	TTCACATCAAAACAACTCCCTT
C TalBK2 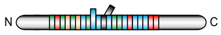 without repeat p12	1	*Os12g29220.1*	nodulin MtN3 family protein (*OsSWEET13*)	267	-6,250	TATAAAGCACCACAACTCCCTT
	2	*Os02g03750.1*	polygalacturonase, putative, expressed	308	-14,889	CATAGAACACCACACCTCCATC
	3	*Os04g54790.1*	ELMO/CED-12 family protein	283	-15,287	TAGAAACCACCACCACTCTCTT
	4	*Os10g30440.1*	expressed protein	222	-15,649	TATAAAACACCACTCCTTCCTT
	5	*Os01g51030.1*	expressed protein	265	-15,930	TATAAAACACCCAAACTCCCCG
D TalBK2 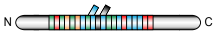 without repeat p9 and p12	1	*Os05g24230.1*	hypothetical protein	142	-15,177	TATAAACCACAAAACTACCTA
	2	*Os01g42860.1*	inhibitor I family protein, putative ,expressed	269	-15,361	AATACACCACACAACTCCATC
	3	*Os11g13710.1*	expressed protein	189	-15,648	TATAAACCACCAAACACCCTC
	4	*Os01g05220.1*	expressed protein	210	-15,859	TCTCCAGCAACCAACTCCCTC
	5	*Os11g05690.3*	amino acid permase family protein	44	-15,900	TCAACAGTACAAAACTCCCTT
E TalBK2 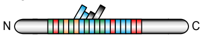 without repeat p9 - 12	1	*Os10g32050.1*	anykrin repeat domain containing protein	20	-9,587	TATAGAGTACAACTCCCTC
	2	*Os06g23300.1*	expressed protein	225	-10,442	CATCGAGCACAACTCCCTC
	3	*Os06g32960.1*	peroxidase precursor, putative	154	-11,881	TATAAAGCACACCTCCGTT
	4	*Os09g19480.1*	retrotransposon, putative	67	-12,938	TATCCAGCCCAACTCCCTG
	5	*Os04g46650.1*	expansin precursor, puative	15	-13,153	TAACCAACACAACTACCTT

Supplemental Figure 10: Target prediction analysis for TalBK2. The analysis is based on TalBK2 binding DNA (**A**) with all repeats participating, (**B**) with the aberrant repeat at position 9 looping out of the repeat array, (**C**) with the aberrant repeat at position 12 looping out the repeat array, (**D**) with both aberrant repeats (positions 9 and12) looping out of the repeat array and (**E**) with repeats at 9 to 12 looping out of the repeat array.

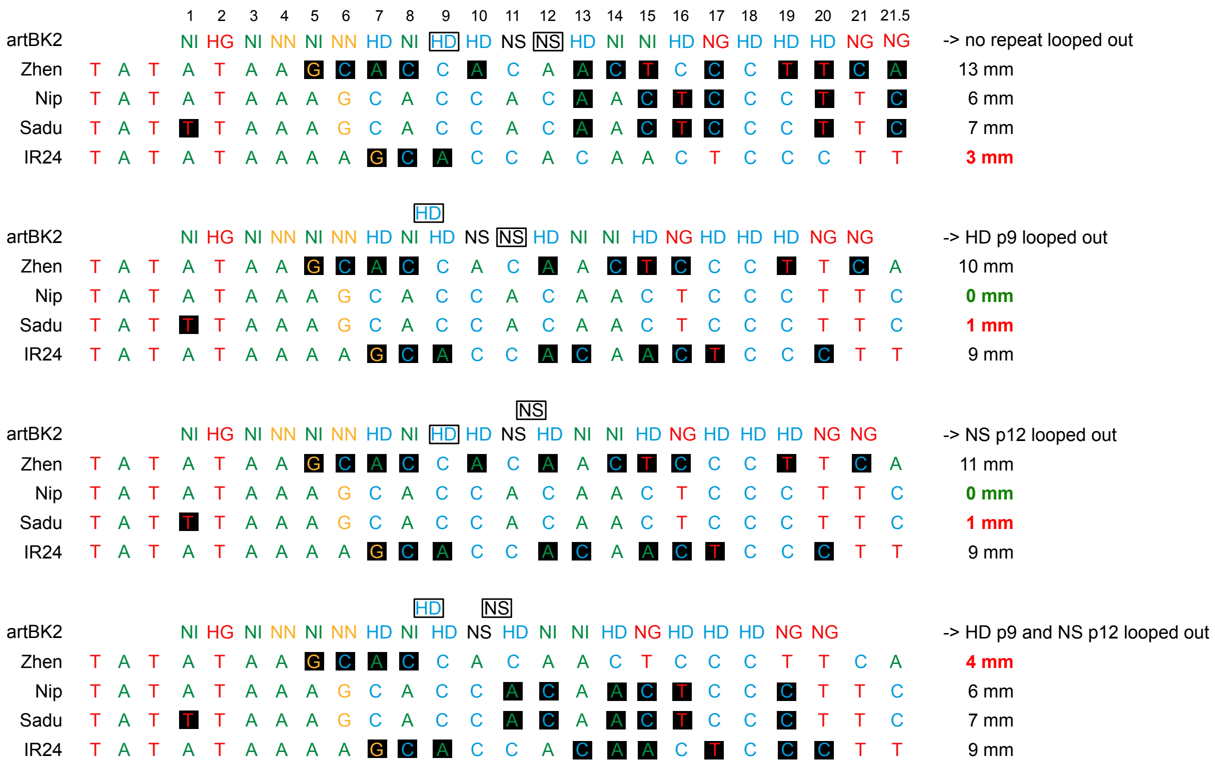

Supplemental Figure 11: Best binding possibilities of TALE artBK2 in the *OsSWEET13* promoter. Shown are the four different rice cultivars Zhenshan (Zhen), Nipponbare (Nip), Sadu Cho (Sadu) and IR24. Aberrant repeats are boxed, deleted positions within the target boxes are indicated by dashed squares. A mismatch of a RVD to the target box is indicated by a black square. Mismatched repeats (mm). The lowest number of mismatches for each rice cultivar is shown in bold. Colour code indicates activity in our GUS assay: green, active; red, not active.

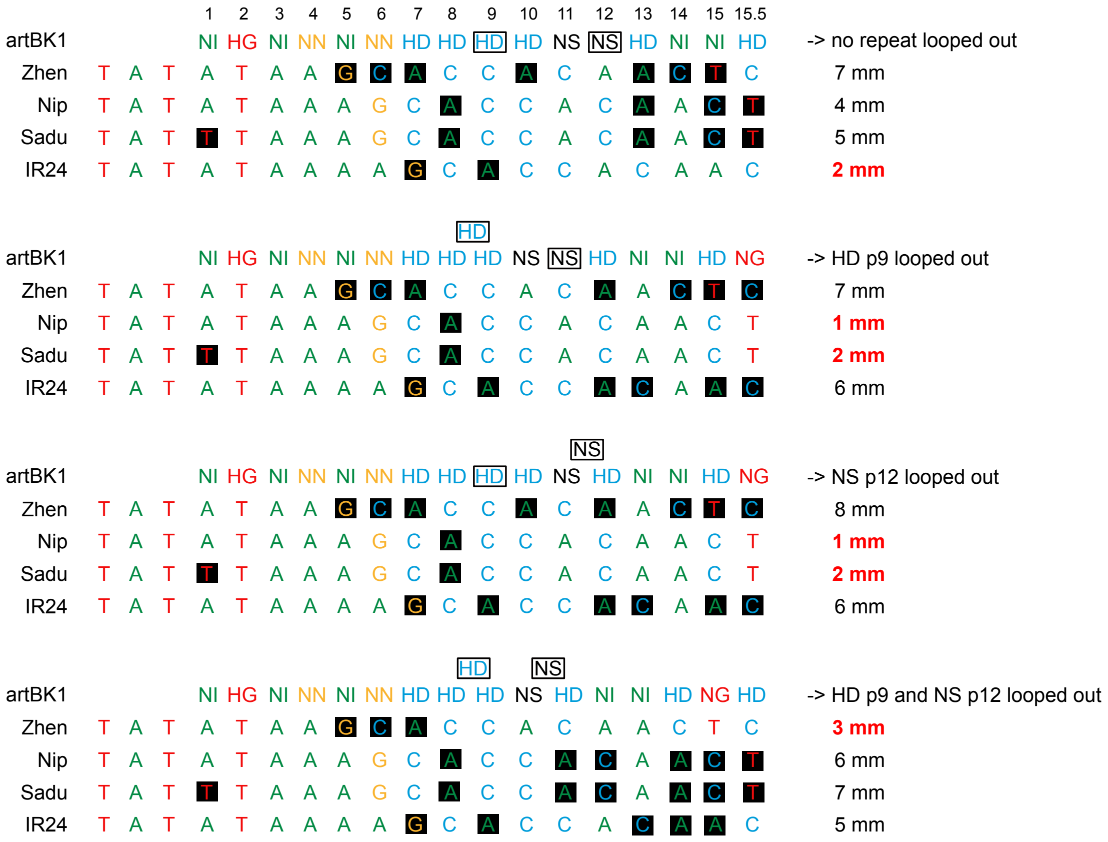

Supplemental Figure 12: Best binding possibilities of TALE artBK1 in the *OsSWEET13* promoter. Shown are the four different rice cultivars Zhenshan (Zhen), Nipponbare (Nip), Sadu Cho (Sadu) and IR24. Aberrant repeats are boxed, deleted positions within the target boxes are indicated by dashed squares. A mismatch of a RVD to the target box is indicated by a black square. Mismatched repeats (mm). The lowest number of mismatches for each rice cultivar is shown in bold. Colour code indicates activity in our GUS assay: green, active; red, not active.

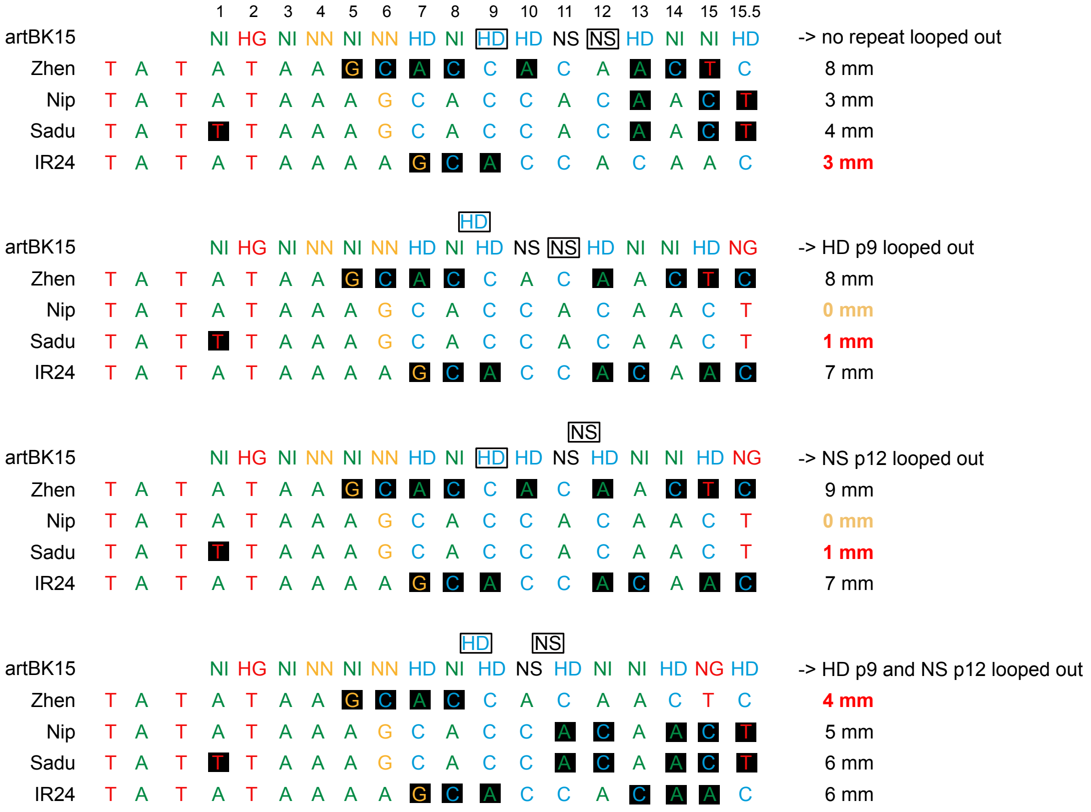

Supplemental Figure 13: Best binding possibilities of TALE artBK15 in the *OsSWEET13* promoter. Shown are the four different rice cultivars Zhenshan (Zhen), Nipponbare (Nip), Sadu Cho (Sadu) and IR24. Aberrant repeats are boxed, deleted positions within the target boxes are indicated by dashed squares. A mismatch of a RVD to the target box is indicated by a black square. Mismatched repeats (mm). The lowest number of mismatches for each rice cultivar is shown in bold. Colour code indicates activity in our GUS assay: yellow, minor activity; red, not active.

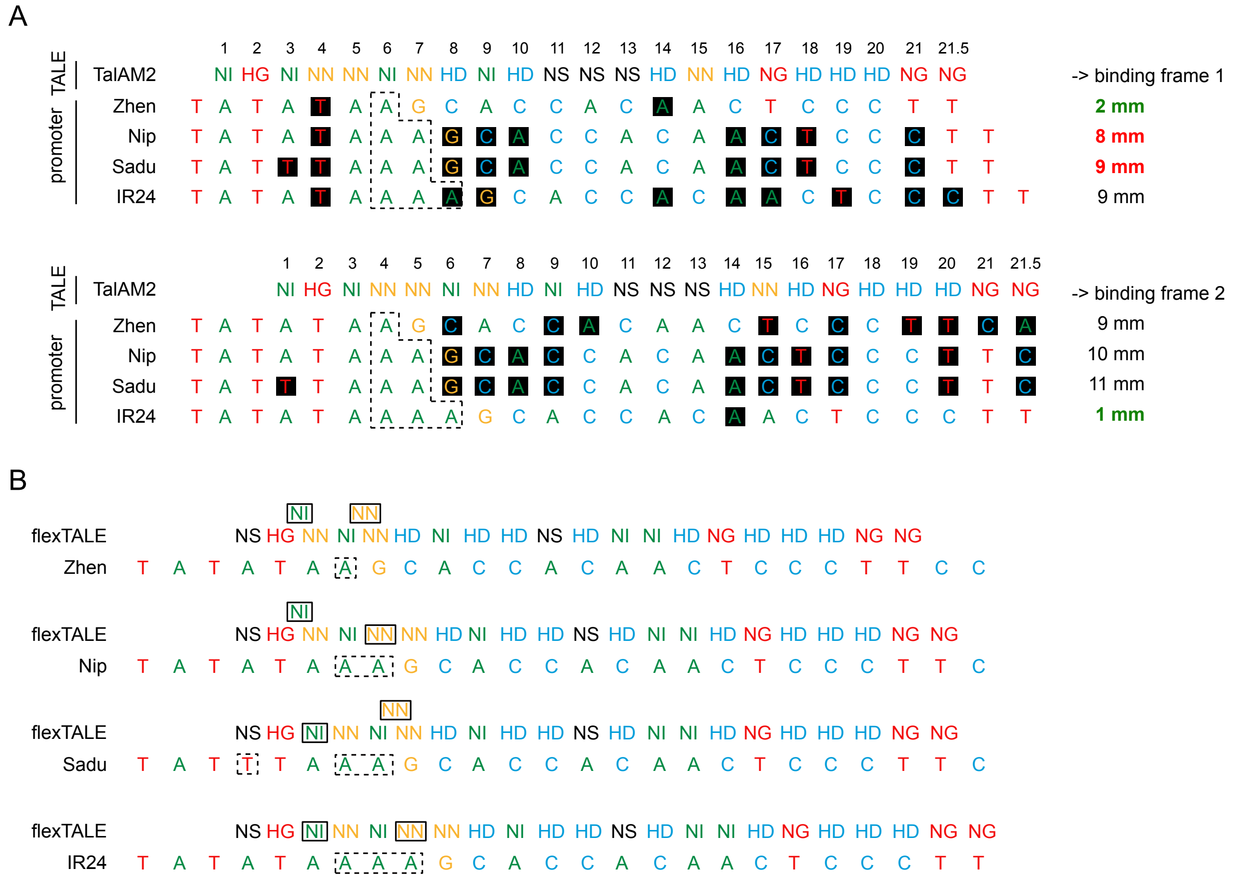

Supplemental Figure 14: Best binding possibilities of TalAM2 and the flexTALE in the *OsSWEET13* promoter. (A) TalAM2. **(B)** flexTALE. Shown are the four different rice cultivars Zhenshan (Zhen), Nipponbare (Nip), Sadu Cho (Sadu) and IR24. Aberrant repeats are boxed, positions affecting the binding of the TALEs within the target boxes are indicated by dashed squares. A mismatch to the target box is indicated by a black square. The lowest number of mismatches for each rice cultivar is indicated in bold. Colour code indicates activity in our GUS assay: green, active; red, not active. Mismatched repeats (mm).

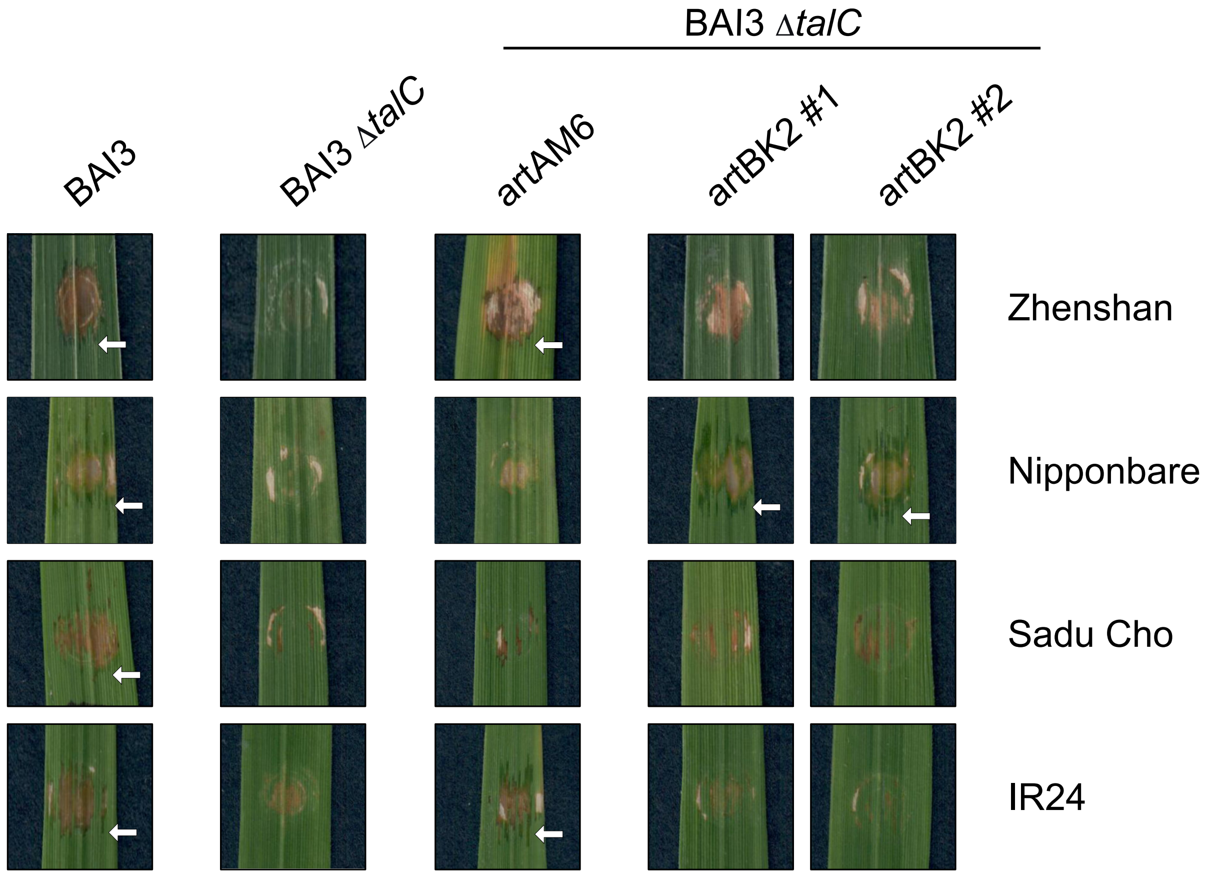

Supplemental Figure 15: TALE artBK2 and artAM6 cause virulence on different rice cultivars. Using a needleless syringe, leaves of 4 week-old rice plants were inoculated with the *Xoo* wild type strain BAI3, the avirulent mutant strain BAI3 Δ*talC*, or with mutant strains containing a plasmid expressing either TALE artAM6 or artBK2 (two tranformants, #1 and #2). Strains were inoculated on the rice cv. Zhenshan, Nipponbare, Sadu Cho and IR24. Disease phenotypes (water-soaking lesions, white arrows) were visible on all four rice cultivars when inoculated with the positive control, the BAI3 wild type strain. This is due to the fact that its major virulence factor, TalC (TalBS1), induces another major susceptibility gene, *OsSWEET14*, and thus is not affected by the variations in the *OsSWEET13* promoter. When inoculated with a BAI3 Δ*talC* strain expressing artAM6, virulence symptoms were observed on Zhenshan and IR24 while a BAI3 Δ*talC* strain expressing artBK2 induced water-soaking lesions on the rice cultivar Nipponbare.

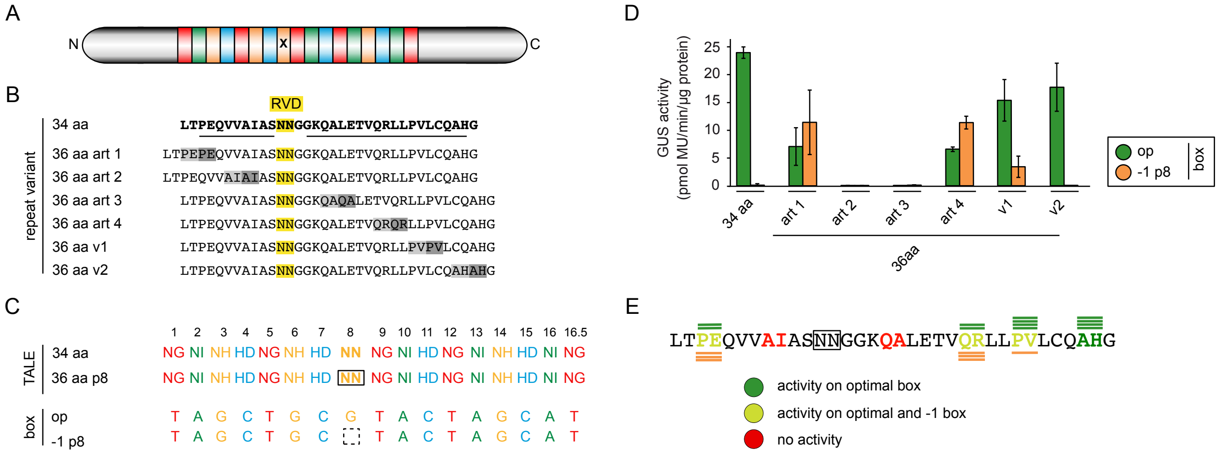

Supplemental Figure 16: Functionality of 36 aa aberrant repeats. (A) TALE setup. The altered repeat is indicated (x). (**B**) Amino acid alignment of used repeat variants. A standard 34 aa repeat is shown in bold with helix-forming residues underlined. RVDs (yellow), duplicated aa (grey). Artificial variants 36 aa art 1 - art 4 were generated by introducing two amino acid duplications at different positions throughout the repeat (duplicated positions: 3-4, 8-9, 17-18, 23-24, respectively). The aberrant repeats 36 aa v1 and v2 are naturally occurring variants (duplicated positions: 27-28, 32-33, respectively). (**C**) RVDs of generated TALEs. Position of the aberrant repeat is boxed. The TALE box was either optimal (op) or a frameshift variant with one nucleotide deleted (-1 p8). (**D**) GUS assay of TALEs on boxes shown in (C). Error bars represent standard deviation (n = 3). (**E**) Tolerance of a TALE repeat towards small duplications. Colour code: green, activity at optimal box (no looping out); light green, activity at optimal and frameshift box (flexible binding, partial looping out), activity strength depends on position of the duplication; red, loss of TALE activity. Number of lines below the aa correspond to relative activity in (D). RVDs (boxed).

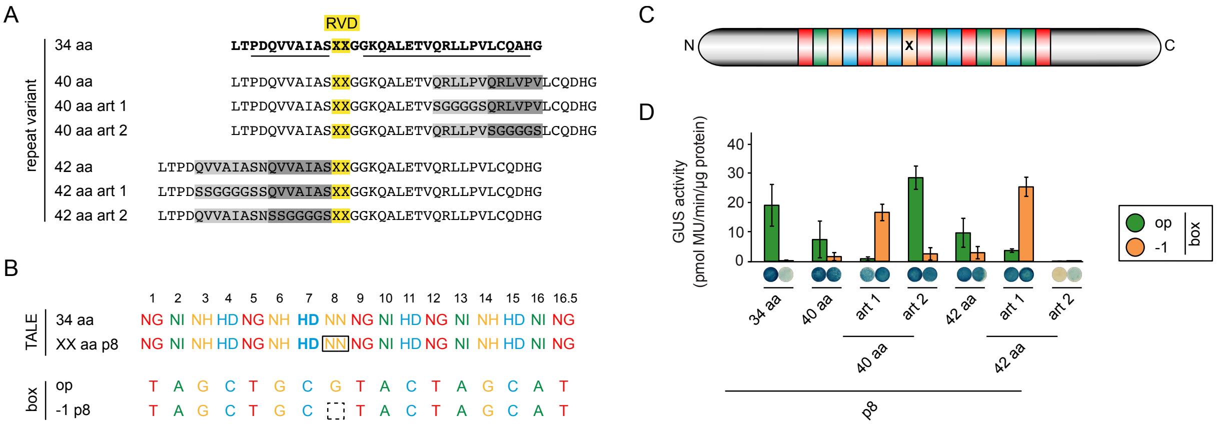

Supplemental Figure 17: Linker swap analysis. (**A**) Amino acid alignment of used repeat variants. A standard 34 aa repeat is shown in bold with helix-forming residues underlined. RVDs (yellow), altered aa (grey). Artificial variants of the aberrant repeats with 40 and 42 aa were generated by swapping the duplicated amino acids with a stretch of serines/glycines (art x). (**B**) RVDs of generated TALEs. The aberrant repeat is boxed. The TALE box was either optimal (op) or a frameshift variant with one nucleotide deleted (-1 p8). (**C**) TALE setup. The altered repeat is indicated (x). (**D**) GUS assay of TALEs on boxes shown in (B). Error bars represent standard deviation (n = 3).

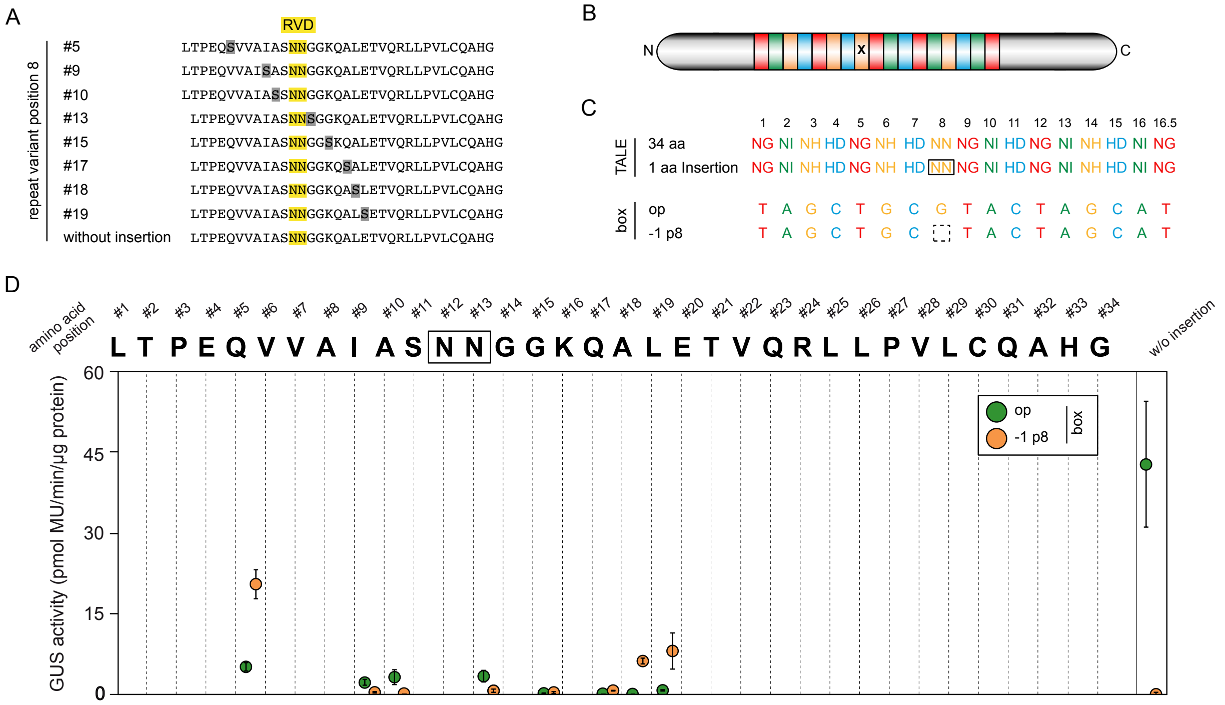

Supplemental Figure 18: Tolerance of a TALE repeat for single amino acid insertions. (**A**) Amino acid alignment of constructed repeat variants. A single serine (S) was inserted at different positions (#) throughout a TALE repeat. RVDs (yellow), inserted aa (grey). (**B**) TALE setup. The altered repeat is indicated (x). (**C**) RVDs of generated TALEs. Position of the aberrant repeat is boxed. TALE box was either optimal (op) or a frameshift variant with a one nucleotide deletion at position 8 (-1 p8). (**D**) GUS assay of TALEs with repeat variants on boxes shown in (C). Error bars represent standard deviation (n = 3).

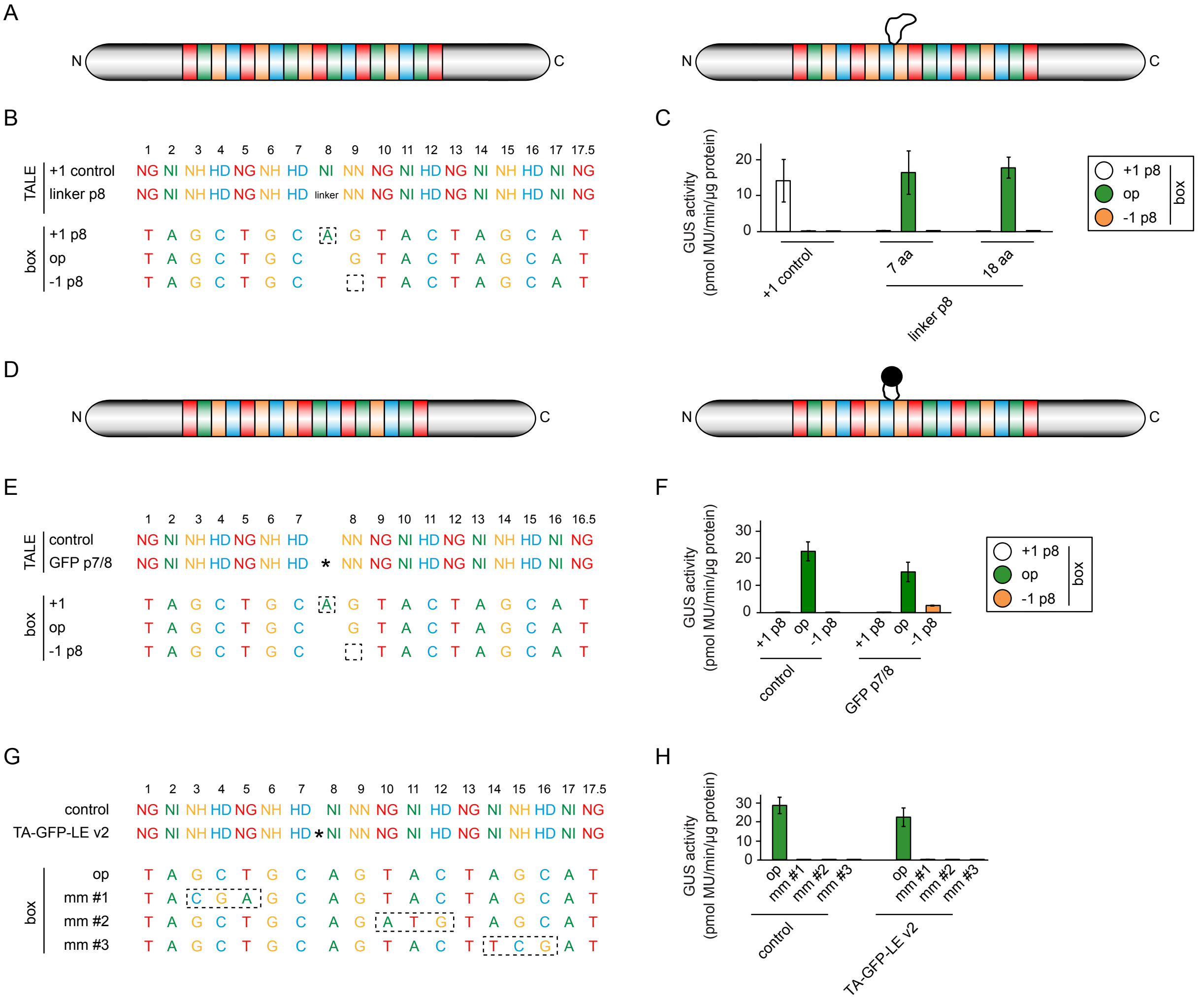

Supplemental Figure 19: Functional domains can be inserted between two TALE repeats. (**A**) and (**D**) TALE setup. Insertions into the repeat array are indicated. (**B**), (**E**) and (**G**) RVDs of constructed TALEs. (**B**) SG linker sequences with 7 or 18 aa were used instead of repeat 8. TALE boxes were optimal (op), or frameshift variants with either one additional nucleotide (+1 p8) or one nucleotide deleted (-1 p8). (**E**) A GFP (*) tag was inserted between repeat 7 and 8. TALE boxes were optimal (op), or frameshift variants with either one additional nucleotide (+1 p8) or one nucleotide deleted (-1 p8). (**G**) TA-GFP-LE v2 was combined with boxes containing mismatches in areas in front of or after the inserted GFP (mm #1-3). (**C**), (**F**) and (**H**) GUS assay of the TALEs on boxes shown in (B), (E) and (G), respectively. Error bars represent standard deviation (n = 3).

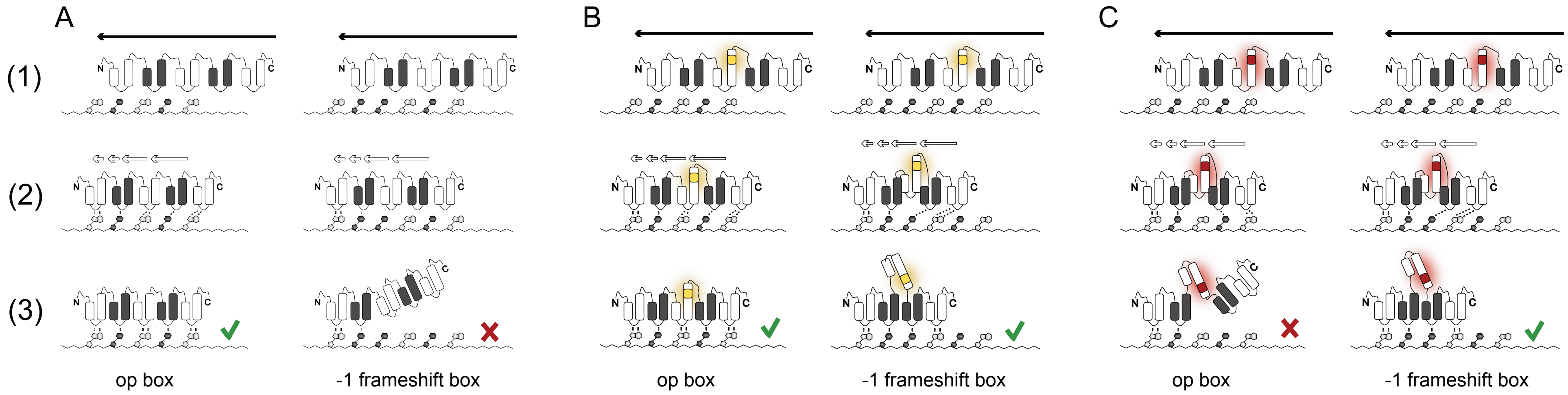

Supplemental Figure 20: Models showing likely dynamic changes in TALE structure during binding. (A) TALE with only 34 aa standard repeats, **(B)** TALE with an aberrant repeat conferring flexible frameshift binding (yellow), or **(C)** TALE with an aberrant repeat with obligatory loop out behaviour (red). **(1)** The TALE moves along the DNA in a one dimensional and non-rotational fashion. **(2)** Upon encountering a potential target sequence, the TALE starts to condense, bringing more and more repeats in sync. **(3)** If enough repeats match, the TALE enters the fully condensed binding mode (green checkmark). If there are too many mismatches present, the TALE relaxes again and resumes the search process (red cross). Aberrant repeats conferring a flexible frameshift binding can participate in DNA-binding, thus behaving like a standard 34 aa repeat, or they can leave the repeat array to allow for the recognition of a -1 frameshift box. Aberrant repeats with an obligatory loop out behaviour cannot participate in DNA-binding and always leave the repeat array, thus they require the presence of a -1 frameshift box with a nucleotide deletion in close proximity to the position of the aberrant repeat.

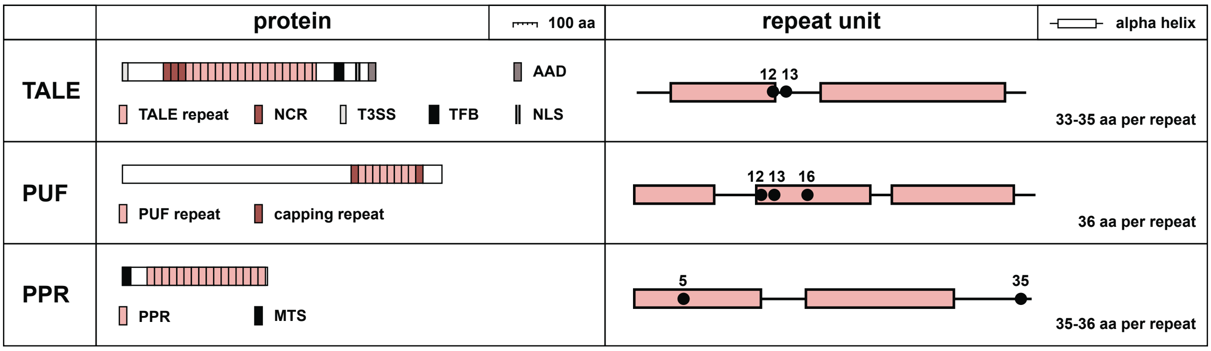

Supplemental Figure 21: Comparison of TALEs, PUFs und PPRs. (Left) Schematic representation of typical TALE, PUF and PPR proteins. Repeats and other motifs of significance are indicated. Non canonical repeats (NCR), signal for T3SS-dependent translocation (T3SS), TFIIAγ binding-site (TFB), nuclear localization signals (NLS), acidic activation domain (AAD), mitochondrial targeting sequence (MTS). **(Right)** Schematic representation showing the secondary structure of a typical repeat unit from a TALE, PUF and PPR protein. Amino acids involved in the specific recognition of nucleotides are indicated by black dots.

Table S1: Oligonucleotides.

sequence	use
TTTGAAGACTTTTACCCCGGAGCAGGTGGTGGCC	repeat overhangs (p1)
TTTGAAGACTTTCAGGCCATGGGCCTGGCACAGCAC	
TTTGAAGACTTCTGACCCCGGAGCAGGTGGTG	repeat overhangs (p2)
TTTGAAGACTTCGGTGTCAGGCCATGGGCCTGGCACAGCAC	
TTTGAAGACTTACCGGAGCAGGTGGTGGCCATCGCC	repeat overhangs (p3)
TTTGAAGACTTGGTGAGGCCATGGGCCTGGCACAGCAC	
TTTGAAGACTTCACCCCGGAGCAGGTGGTGGCC	repeat overhangs (p4)
TTTGAAGACTTGAGTCAGGCCATGGGCCTGGCACAGCAC	
TTTGAAGACTTACTCCGGAGCAGGTGGTGGCCATC	repeat overhangs (p5)
TTTGAAGACTTATGGGCCTGGCACAGCACCGG	
TTTGAAGACTTCCATGGCCTGACCCCGGAGCAGGTGG	repeat overhangs (p6/stop)
TTTGAAGACTTTCCAGCGCCTGCTTGCC	
ATGGCCACCACCTGCTCCGG	mutagenesis RVD (universal fw)
CGCCAGCCACGATGGCGGCAAGCAGGCGCTGGAGACG	mutagenesis RVD (HD rv)
CGCCAGCAATGGCGGCAAGCAGGCGCTGGAGA	mutagenesis RVD (N* rv)
CGCCAGCAATGGCGGCGGCAAGCAGGCGCTGGAGACG	mutagenesis RVD (NG rv)
CGCCAGCAATATTGGCGGCAAGCAGGCGCTGGAGACG	mutagenesis RVD (NI rv)
CGCCAGCAATAACGGCGGCAAGCAGGCGCTGGAGACG	mutagenesis RVD (NN rv)
GCCATGGGCCTGGCACAGCA	37 aa v1 repeat module (rv)
CCCCATGGCCTGAAAGTCTTCAAA	37 aa v1 repeat module (p1 fw)
CCCCATGGCCTGACACCGAAGTCTT	37 aa v1 repeat module (p2 fw)
CCCCATGGCCTCACCAAGTCTTCAAA	37 aa v1 repeat module (p3 fw)
CCCCATGGCCTGACTCAAGTCTTCAAA	37 aa v1 repeat module (p4 fw)
GTGCAGCGGCTGTTGCCGGTGCTGTGCC	37 aa v2 repeat module (fw)
CCGCTGCACCGTCTCCAGCGCCTGC	37 aa v2 repeat module (rv)
CCGGTGCTGTGCCAGGCCCATGGC	36 aa v1 repeat module (fw)
CACCGGCAACAGCCGCTGCACCGTC	36 aa v1 repeat module (rv)
GCCCATGCCCATGGCCTGACACCGAAG	36 aa v2 repeat module (fw)
CTGGCACAGCACCGGCAACAGCCGC	36 aa v2 repeat module (rv)
AAGCAGGCGCTGGAGACGGTGCA	36 aa v3 repeat module (fw)
GCCGCCGCCATCGTGGCTG	36 aa v3 repeat module (rv)
CCGCATGATCTGACACCGAAGTCTTC	35 aa v1 repeat module (fw)
GGCCTGGCACAGCACCGGCAA	35 aa v1 repeat module (rv)
CCGCCGTACGGCCTGACACCGAAGTCTTC	35 aa v2 repeat module (fw)
CTGGCACAGCACCGGCAACAGCCGCTG	35 aa v2 repeat module (rv)
TTTGAAGACTTTTACCCCGGACCAGGTGGTGGCC	30 aa repeat module (p1 fw)
TTTGAAGACTTTCAGGCACAGCACCGGCAACAGC	30 aa repeat module (p1 rv)
TTTGAAGACTTCTGATCCCGGACCAGGTGGTGGC	30 aa repeat module (p2 fw)
TTTGAAGACTTCGGTGTCAGGCACAGCACCGGCAACAGC	30 aa repeat module (p2 rv)
TTTGAAGACTTACCGGACCAGGTGGTGGCCATCG	30 aa repeat module (p3 fw)
TTTGAAGACTTGGTGAGGCACAGCACCGGCAACAGC	30 aa repeat module (p3 rv)
TTTGAAGACTTCACCCCGGACCAGGTGGTGGCCA	30 aa repeat module (p4 fw)
TTTGAAGACTTGAGTCAGGCACAGCACCGGCAACAGC	30 aa repeat module (p5 rv)
GTGCTGTGCCAGGCCCATGG	28 aa repeat module (fw)
CGTCTCCAGCGCCTGCTTGC	28 aa repeat module (rv)
CGGCAACAGCCGCTGCACCGTCTCCAAAGTCTTCG	40 aa AV (universal rv)
GTACAGCGGCTGGTGCCGGTGCTGTGCCAGGTGA	40 aa AV (AB fw)
CAGGTGGTGGCCATCGCCAG	36 aa art 1 repeat module (fw)
CTCCGGCTCCGGTAAGTCTTC	36 aa art 1 repeat module (rv)
GCCATCGCCAGCAATAACGGTG	36 aa art 2 repeat module (fw)
GATGGCCACCACCTGCTCC	36 aa art 2 repeat module (rv)
CAGGCGCTGGAGACGGTGCA	36 aa art 3 repeat module (fw)
CGCCTGCTTGCCACCGTTATTG	36 aa art 3 repeat module (rv)
CAGCGGCTGTTGCCGGTGC	36 aa art 4 repeat module (fw)
CCGCTGCACCGTCTCCAGC	36 aa art 4 repeat module (rv)
CACCGGCAACAGCCGCTGCACCGTCTCCAGCGCC	48 aa repeat module (fw)
CAGCGGCTGGTGCCGGTGCTGTGCCAGGCCCATGGC	48 aa repeat module (rv)
CAGGTGGTGGCCATCGCCAGCAATAACGGTGGCAA	42 aa art 1 repeat module (fw)
CGAGCTGCCACCGCCACCCGAGCTCTCCGGTAAGTCTT	42 aa art 1 repeat module (rv)
ATTGCTGGCGATGGCCACCACCTGCTCCGGTA	42 aa art 2 repeat module (fw)
AGCTCGGGTGGCGGTGGCAGCAATAACGGTGGCA	42 aa art 2 repeat module (rv)
AGCGGTGGCGGTGGCAGCCAGCGGCTGTTG	40 aa art 1 repeat module (fw)
CACCGTCTCCAGCGCCTGCTTGCCACC	40 aa art 1 repeat module (rv)
AGCGGTGGCGGTGGCAGCCTGTGCCAGGCC	40 aa art 2 repeat module (fw)
CACCGGCAACAGCCGCTGCACCGTCTCCAGCG	40 aa art 2 repeat module (rv)
TTTGAAGACTTCTGAGCTCGGGTGGCGGT	7aa linker repeat module (p2 fw)
TTTGAAGACTTCGGTGTCAGGCTGCCACCGCCACCC	7aa linker repeat module (p2 rv)
TTTGAAGACTTCTGAGCTCGGGTGGCGGTAGCGGCGGTTCGGGCAGCGGC	18aa linker repeat module (p2 fw)
TTTGAAGACTTCGGTGTCAGCGAGCTACCGCTGCCACCGCCGCTGCCCGA	18aa linker repeat module (p2 rv)
TTTGAAGACTTCTGAGCTCGGGTGGCGGT	7aa linker repeat module (p2.1 fw)
TTTGAAGACTTGCTGCCACCGCCACCCGA	7aa linker repeat module (p2.1 rv)
TTTGAAGACTTCAGCATGGTGAGCAAGGGC	GFP-tag repeat module (p2.2 fw)
TTTGAAGACTTCACCCTTGTACAGCTCGTCC	GFP-tag repeat module (p2.2 rv)
TTTGAAGACTTCAGCTATCCTTATGACGTGCCTGACTATGCCAG	HA-tag repeat module (p2.2 fw)
TTTGAAGACTTCACCAGGTCCTCCCAGGCTGGCATAGTCA	HA-tag repeat module (p2.2 rv)

TTTGAAGACTTGGTGGCTCGAGCGGTGGCAGC	7aa linker repeat module (p2.3 fw)
TTTGAAGACTTCGGTGTCAGGCTGCCACCGCTC	7aa linker repeat module (p2.3 rv)
CAGGTGGTGGCCATCGCCAGCAATAACGGTGGCAA	3NN 7 aa #4 repeat module (fw)
GCTGCCACCGCCACCCGAGCTCTCCGGTAAGTCTT	3NN 7 aa #4 repeat module (rv)
GTGGTGGCCATCGCCAGCAA	3NN 1 aa #5 repeat module (fw)
CGACTGCTCCGGTAAGTCTTC	3NN 1 aa #5 repeat module (rv)
GTGGTGGCCATCGCCAGCAATAACGGTGGCAAGC	3NN 7 aa #5 repeat module (fw)
GCTGCCACCGCCACCCGAGCTCTGCTCCGGTAAGT	3NN 7 aa #5 repeat module (rv)
GCCAGCAATAACGGTGGCAAGCAGGCGCTGG	3NN 1/7 aa #9 repeat module (fw)
CGAGATGGCCACCACCTGCTCCGGTAAG	3NN 1 aa #9 repeat module (rv)
GCTGCCACCGCCACCCGAGCTGATGGCCACCA	3NN 7 aa #9 repeat module (rv)
AGCAATAACGGTGGCAAGCAGGCGCTGGAGACG	3NN 1/7 aa #10 repeat module (fw)
CGAGGCGATGGCCACCACCTGCTC	3NN 1 aa #10 repeat module (rv)
GCTGCCACCGCCACCCGAGCTGGCGATGGCCA	3NN 7 aa #10 repeat module (rv)
AGCTCGGGTGGCGGTGGCAGCAATAACGGTGGCA	3NN 7 aa #11 repeat module (fw)
GCTGGCGATGGCCACCACCTGCTCCGGT	3NN 7 aa #11 repeat module (rv)
TCGGGTGGCAAGCAGGCGCTGGAGACG	3NN 1 aa #13 repeat module (fw)
AGCTCGGGTGGCGGTGGCAGCGGTGGCAAGCA	3NN 7 aa #13 repeat module (fw)
GTTATTGCTGGCGATGGCCACCACCTGCTCC	3NN 1/7 aa #13 repeat module (rv)
TCGAAGCAGGCGCTGGAGACGGTG	3NN 1 aa #15 repeat module (fw)
AGCTCGGGTGGCGGTGGCAGCAAGCAGGCGCT	3NN 7 aa #15 repeat module (fw)
GCCACCGTTATTGCTGGCGATGGCCACCACC	3NN 1/7 aa #15 repeat module (rv)
TCGGCGCTGGAGACGGTGCAGCGG	3NN 1 aa #17 repeat module (fw)
AGCTCGGGTGGCGGTGGCAGCGCGCTGGAGAC	3NN 7 aa #17 repeat module (fw)
CTGCTTGCCACCGTTATTGCTGGCGATGGCCAC	3NN 1/7 aa #17 repeat module (rv)
TCGCTGGAGACGGTGCAGCGGCTGTTG	3NN 1 aa #18 repeat module (fw)
AGCTCGGGTGGCGGTGGCAGCCTGGAGACGGT	3NN 7 aa #18 repeat module (fw)
CGCCTGCTTGCCACCGTTATTGCTGGCGATGGCC	3NN 1/7 aa #18 repeat module (rv)
TCGGAGACGGTGCAGCGGCTGTTGCCG	3NN 1 aa #19 repeat module (fw)
AGCTCGGGTGGCGGTGGCAGCGAGACGGTGCA	3NN 7 aa #19 repeat module (fw)
CAGCGCCTGCTTGCCACCGTTATTGCTGGCGATG	3NN 1/7 aa #19 repeat module (rv)
AGCTCGGGTGGCGGTGGCAGCACGGTGCAGC	3NN 7 aa #20 repeat module (fw)
CTCCAGCGCCTGCTTGCCACCGTTATTGCTG	3NN 7 aa #20 repeat module (rv)
AGCTCGGGTGGCGGTGGCAGCCAGCGGCTGTTGCCG	3NN 7 aa #22 repeat module (fw)
CACCGTCTCCAGCGCCTGCTTGCCACCG	3NN 7 aa #22 repeat module (rv)
AGCTCGGGTGGCGGTGGCAGCCTGTTGCCGGT	3NN 7 aa #24 repeat module (fw)
CCGCTGCACCGTCTCCAGCGCCTGCTTGCCA	3NN 7 aa #24 repeat module (rv)
TCGTTGCCGGTGCTGTGCCAGGCCCATGG	3NN 1 aa #25 repeat module (fw)
AGCTCGGGTGGCGGTGGCAGCTTGCCGGTGCT	3NN 7 aa #25 repeat module (fw)
CAGCCGCTGCACCGTCTCCAGCGCCTGCTTG	3NN 1/7 aa #25 repeat module (rv)
AGCTCGGGTGGCGGTGGCAGCCCGGTGCTGTG	3NN 7 aa #26 repeat module (fw)
CAACAGCCGCTGCACCGTCTCCAGCGCCTGC	3NN 7 aa #26 repeat module (rv)
AGCTCGGGTGGCGGTGGCAGCCTGTGCCAGGCC	3NN 7 aa #28 repeat module (fw)
CACCGGCAACAGCCGCTGCACCGTCTCCAGCG	3NN 7 aa #28 repeat module (rv)
AGCTCGGGTGGCGGTGGCAGCTGCCAGGCCCATGG	3NN 7 aa #29 repeat module (fw)
CAGCACCGGCAACAGCCGCTGCACCGTCTCCAGCGC	3NN 7 aa #29 repeat module (rv)
AGCTCGGGTGGCGGTGGCAGCGCCCATGGCCT	3NN 7 aa #31 repeat module (fw)
CTGGCACAGCACCGGCAACAGCCGCTGCACC	3NN 7 aa #31 repeat module (rv)
TTTGGTCTCACACCAGGGATGTCTACTGCAGGTG	1 kbp *OsSWEET13* promoter (fw)
TTTGGTCTCACACCCATTTTTGTGTGCTAAAAGGGGGGTA	1 kbp *OsSWEET13* promoter (rv)
TTTGGTCTCACCTTAGATTCGATTAAAAATAAATTGTATG	TALE box (universal rv)
TTTGGTCTCACACCTTAGCTGCAGTACTAGCATATTCTTTCTTGTATATAACTTTGTCC	TALE box “op” (fw)
TTTGGTCTCACACCTTAGTGCAGTACTAGCATATTCTTTCTTGTATATAACTTTGTCC	TALE box “op -1 p4” (fw)
TTTGGTCTCACACCTTAGCTCAGTACTAGCATAATTCTTTCTTGTATATAACTTTGTCC	TALE box “op -1 p6” (fw)
TTTGGTCTCACACCTTAGCTGAGTACTAGCATAATTCTTTCTTGTATATAACTTTGTCC	TALE box “op -1 p7” (fw)
TTTGGTCTCACACCTTAGCTGCGTACTAGCATATTCTTTCTTGTATATAACTTTGTCC	TALE box “op -1 p8” (fw)
TTTGGTCTCACACCTTAGCTGCATACTAGCATATTCTTTCTTGTATATAACTTTGTCC	TALE box “op -1 p9” (fw)
TTTGGTCTCACACCTTAGCTGCAGTATAGCATATTCTTTCTTGTATATAACTTTGTCC	TALE box “op -1 p12” (fw)
TTTGGTCTCACACCTTAGTGCAGTATAGCATTATTCTTTCTTGTATATAACTTTGTCC	TALE box “op -2 p4/12” (fw)
TTTGGTCTCACACCTTAGCTAGTACTAGCATTAATTCTTTCTTGTATATAACTTTGTCC	TALE box “op -2 p6/7” (fw)
TTTGGTCTCACACCTTAGCTGGTACTAGCATTATTCTTTCTTGTATATAACTTTGTCC	TALE box “op -2 p7/8” (fw)
TTTGGTCTCACACCTTAGCTGCTACTAGCATTATTCTTTCTTGTATATAACTTTGTCC	TALE box “op -2 p8/9” (fw)
TTTGGTCTCACACCTTAGCTGTACTAGCATGTATTCTTTCTTGTATATAACTTTGTCC	TALE box “op -3 p7/8/9” (fw)
TTTGGTCTCACACCTTAGCTGGGGGGGTAGCATTTCTTTCTTGTATATAACTTTGTCC	TALE box “G6 op” (fw)
TTTGGTCTCACACCTTAGCTGGGGGGTAGCATATTCTTTCTTGTATATAACTTTGTCC	TALE box “G6 -1” (fw)
TTTGGTCTCACACCTTAGCTGGGGGTAGCATTATTCTTTCTTGTATATAACTTTGTCC	TALE box “G6 -2” (fw)
TTTGGTCTCACACCTTAGCTGGGGTAGCATGTATTCTTTCTTGTATATAACTTTGTCC	TALE box “G6 -3” (fw)
TTTGGTCTCACACCTTAGCTGGGTAGCATCGTATTCTTTCTTGTATATAACTTTGTCC	TALE box “G6 -4” (fw)
TTTGGTCTCACACCTTAGCTGGTAGCATCCGTATTCTTTCTTGTATATAACTTTGTCC	TALE box “G6 -5” (fw)
TTTGGTCTCACACCTTAGCTGTAGCATCGCGTATTCTTTCTTGTATATAACTTTGTCC	TALE box “G6 -6” (fw)
TTTGGTCTCACACCTATAGAGCACCAACAACTCCCTTTTCTTTCTTGTATATAACTTTGTCC	TALE box “BK op” (fw)
TTTGGTCTCACACCTATAGAGCACAACAACTCCCTTTTCTTTCTTGTATATAACTTTGTCC	TALE box “BK -1 p9” (fw)
TTTGGTCTCACACCTATAGAGCACCACAACTCCCTTTTCTTTCTTGTATATAACTTTGTCC	TALE box “BK -1 p12” (fw)
TTTGGTCTCACACCTATAGAGCACACAACTCCCTTTTCTTTCTTGTATATAACTTTGTCC	TALE box “BK -2 p9/12” (fw)
TTTGGTCTCACACCTATAGAGCACAACTCCCTTTTCTTTCTTGTATATAACTTTGTCC	TALE box “BK -4 p9-12” (fw)
TTTGGTCTCACACCTTACGAGCAGTACTAGCATTTCTTTCTTGTATATAACTTTGTCC	TALE box “mm #1” (fw)
TTTGGTCTCACACCTTAGCTGCAGATGTAGCATTTCTTTCTTGTATATAACTTTGTCC	TALE box “mm #2” (fw)
TTTGGTCTCACACCTTAGCTGCAGTACTTCGATTTCTTTCTTGTATATAACTTTGTCC	TALE box “mm #3” (fw)

Table S2: TALE classes with aberrant repeats.

class	RVD sequence	strain
	42 aa, RVD NI	
TalAQ	HD HD NN NN NI NG HD NS HG HD NG N* HD	PXO142 (pseudogene)
	HD HD NN NN NI NG HD S* HG HD NG N* NG HD HD N* NI NI NN HD HI ND HD NG NN HG N*	PXO61, K1, AUST2013, SK2-3, IXO704, KXO85, K2, PXO71, IX-280, PXO421, XF89b, PXO404, PXO524, JW11089, PXO513, PXO602, T7133, K-74
	-- -- -- -- -- -- -- -- HG HD NG N* NG HD HD N* NI NI NN HD HI ND HD NG NN HG N*	PXO602 (pseudogene)
	HD HD NN NN NG NG HD NS HG HD NG N* HD HD HD N* NN NI NN HD HI ND HD HG NN HG N*	IXO1088, PXO79, ICMP3125, PXO99
	HD HD NN NN NS NG HD S* HG HD NG N* HD HD HD N* NN NI NN HD HM ND HD HG AN HG N*	PXO145
	HD HD NN NN NS NG HD S* HG HD NG N* HD HD HD N* NN NI NN HD HI ND HD HG NN HG N*	PXO563, K3, K3a, XM9, ScYc-b, PXO83, PXO236, JL25, JP01, PXO211, MAFF311018, PXO86, YC11
	-- HD HD NN NS NG HD S* HG HD NG N* HD HD HD N* NN NI NN HD HI ND HD HG NN HG N*	AH28
	-- -- -- NN NS NG HD S* HG HD NG N* HD HD HD N* NN NI NN HD HI ND HD HG NN HG N*	JL28
	40 aa, RVD N*	
TalDS	-- -- -- -- -- -- -- -- -- HD HD HD NS N* N* HD HD NS NS NN NN NI NG NN NI N* NS N*	DY89031 (J18)
	-- -- -- NI HG NI NI NS HD HD HD HD NS N* N* HD HD NS NS NN NN NI NG NN NI N* NS N*	PXO211
TalAC	-- -- NI HG NI NI NS HD NN HD HD HD NS N* N* HD HD NS NS NN NN NI NG NN NI N* NS N*	PXO145, MAFF311018, PXO2684, PXO83, PXO236, KXO85, PXO524, JW11089, K1, PXO86, K2, PXO1865, JP01
	-- -- NI HG NI NI NS HD NN HD HD HD NS N* N* HD HD NS NS NN NN NI NG NN HD NS NN NS N* NS N*	AUST2013
	-- -- NI HG NI NI NS HD NN HD HD HD NS N* N* HD HD NS NS NN N*	KACC10331
TalDV	-- -- NI HG NI NI NS HD NN HD HD HD NS HD N* NI HD HD NN NS NN NN NG NN HD N* NS NS N*	PXO602, PXO563, HuN37, T7133
TalBH	NI HG NI HG NI NI NS HD NN HD HD HD NG HD N* NI HD HD NN NS HD NN NN NG NN HD NG NS N*	IXO704
	NI HG NI HG NI NI NI HD NN HD HD HD NG HD N* NI HD HD NN NS NI NN NN NG NN HD N* NS N*	PXO421, PXO364, PXO142, PXO61 (pseudogene), YC11, JL28, ScYc-b, JL25, JL33, PXO513, PXO404, K3a, AH28 (pseudogene)
n/a	NI HG NI HG NI NI NI HD NN HD HD HD NG HD N* NI HD HD NN NI NN NN NG NN HD N* NS N*	K-74
TalFX	NI HG NI HG NI NI NI HD NN HD HD HD NG HD N* NI HD NN NS NI NN NN NG NN NN NG NN HD N* NS N*	XM9
TalEQ	NI HG NI HG NI NI NI HD NN HD HD HD NG HD N* HD HD NN NS NI NN NN NG NN NN NN NG NN HD N* NS N*	XF89b

	40 aa, RVD NN	
TalAS	-- -- -- -- -- -- -- NI HG NI NN NS HD NN HD HG HD NI NI NN NI HD HD HD HG NN NN HD NS NN HD N* NS N*	IX-280, PXO79, SK2-3, IXO1088
	-- -- -- -- -- -- -- NI HG NI NI HG HD NN HD HD HD NI NS NN NS HD HD HD HG NN NN HD NS NN HD N* NS N*	AUST2013
	-- -- -- -- -- -- -- NI HG NI NI HG HD NN HD HD HD NI NI NN NI HD HD HD HG NN NN HD NS NN HD N* NS N*	PXO83, PXO211, PXO86, PXO145
	-- -- -- -- -- -- -- NI HG NI NI HG HD NN HD HD HD NI NI NN NI HD HD HD HG NN NN HD NS NN HD N* NS N*	KXO85, JW11089, K1
	-- -- -- -- -- -- -- NI HG NI NI HG HD NN HD HD HD NI NI NN NI HD HD HD HG NN NN HD NS NN HD N* NN N*	K2
	-- -- -- -- -- -- -- NI HG NI NS HG HD NN HD HD HD NI NI NN NI HD HD HD HG NN NN HD NS NN HD NG NS N*	PXO71
	-- -- -- -- -- -- -- NI HG NI NI HG HD NN HD HD HD NI NI NN NI HD HD HD HG NN NN HD NS NN HD NG NS N*	JP01, PXO61, PXO524, JL33, HuN37, PXO563, K3a, PXO142, MAFF311018, PXO602, YC11, T7133, AH28, K-74
	-- -- -- -- -- -- -- NI HG NI NI HG HD NN HD HD HD NI NI NN NI HD NS HD HG NN NN HD NS NN HD NG NS N*	XM9
	-- -- -- -- -- -- -- -- -- -- -- -- -- NN HD HD HD NI NI NN NI HD HD HD HG NN NN HD NS NN HD NG NS N*	PXO71 (pseudogene)
	NI HG NI NI NS HD NN HD HG NI NI HG HD NN HD HD HD NI NI NN NI HD HD HD HG NN NN HD NS NN HD N* NS N*	KACC10331
TalER	-- -- -- -- -- -- -- NI HG NI NI HG HD NN HD HD HD NI NI NN NI HD NG NI NN NN HD NS NN HD NG NS N*	XF89b
	-- -- -- -- -- -- -- NI HG NI NI HG HD NN HD HD HD NI NI NN	JL28 (pseudogene)
	37 aa v1, RVD NG	
TalGB	NI HD NI HD NI NG HD NG NG NG NI NI NI NI HD NG	Xac29-1, Xcc49
	NI HD NI HD NI NG HD NG NG NG NI NI NI NI HD NG	306, mf20, A306, LH201, jx5, UI7, FB19, LL074-4, MN11, MN10, jx4, LJ207-7, MN12, NT17, gd2, UI6, LH276, BL18, gd3, jx-6, 5208, gxlp16
	NI HD NI HD NI NG HD NG NG NG NI NI NI NI NI NG	03-1638-1-1
	NI HD NI HD NI NG HD NG NG PG	Xcc29-1 (pseudogene)
	NI HD NS NG NI NG NS NG NG	LMG7439 (pseudogene)
	37 aa v2, RVD HD	
TalGA	NI HD NI HD NI NG N* NI N* NI HD HD NI HD NG	Xac29-1, Xcc49
	36 aa v1, RVD HD (p10, TalFL) or HD and NS (p9 and p12, TalBK)	
TaLFL	NI NG NI NI NI NN HD NI HD HD NG NI NI NS NI HD NS NN HD HD NG N*	AUST2013, IXO704
TalBK	NI HG NI NN NI NN HD NI HD HD NS NS HD NI NI HD	PXO35
	NI HG NI NN NI NN HD NI HD HD NS NS HD NI NI HD NG HD HD HD NG NG	PXO142, PXO61, PXO513, PXO404, PXO421, PXO364, XM9
	NI HG NI NN NI NN HD HD HD HD NS NS HD NI NI HD NG HD HD HD NG NG	JW11089, K1, K2, KXO85, DY89031 (J18)
	NI HG NI NN NI NN HD HD HD HD NS NS HD NI NI HD	KACC10331 (pseudogene)

	36 aa v2, RVD NI	
TalBV	NN HD NI NI NN HA NN NS NI NI HD HA HA HA HD HD HD HA HD N*	CFBP2286
	NN HD NI NI NN HA NN NS NS NI HD HA HA HA HD HD HD HA HD N*	BLS256, GX01, 0-9
	NN HD NI NI NN HA NN NS NS NI HD HA HA HA HD HD HD HA HD N*	L8, RS105, B8-12, BLS279
	NN HD NI NI NN HA NN NS NS NI HD HA HA HA HD HD HD NI HD N*	BXOR1
	36 aa v3, RVD NI	
TalHR	ND HS HD NG HD HD NG HD HD NG NG HD NI HD NR HD HD NG NI NG NG NG	CFBP4691
	30 aa, RVD NN or NI	
TalBG	NI NN NI HD NN NG HD NN HD HG HD HG HG HD HD NG	RS105, L8, B8-12, BLS279,
	NI NN HN NN NI NG HD NN HD HG HD HG HG HD HD NG	CFBP2286, BLS256
	28 aa, RVD NG or HG	
TalAI	-- -- -- -- NS NG NG NG NG NG HD HD HD NN HD NG HD HD HD HD H*	PXO211
	-- -- -- NS NG NG NG NG NG NG HD HD HD NN HD NG HD HD HD HD H*	PXO145, PXO83, PXO86, IXO1088
	-- -- -- NS NN NG NG NG NG NG HD HD HD NN HD NG HD HD HD HD H*	PXO236
	-- -- -- NS HD NG NG NG NG NG HD HD HD NN HD NG HD HD HD HD H*	PXO524, ICMP3125
	-- -- -- NS HD NG NG NG NG NG HD HD HD NN HD NG HD HD HD HD N*	PXO99, PXO79
	-- -- -- NS HD NG NG NG NG NG HD HD HD NN HD NG HD NI HD HD H*	CIAT
	-- -- -- NS HD NG NG NG NG NG HD HD HD NN HD NG HD NI HD NN N*	PXO86, PXO145, PXO83, IXO1088
	-- -- -- NS HD NG NG NG NG HD HD HD HD NN HD NG HD NI HD NN N*	PXO142, ScYc-b, HuN37, YC11, XF89b, PXO61, K3a, PXO563, PXO602, K3, XM9, T7133, K74
	-- -- -- NS HD NG NG NG NG HD HD HD HD NN HD NG HD HD HD NN H*	MAFF311018, JP01
	-- -- -- NS NG NG NG NG NG HD HD HD HD NN HD NG HD HD HD NN H*	PXO282
	-- -- -- -- -- -- -- -- NS HD NG HD HD NN HD NG HD HD HD NN H*	AUST2013
	-- -- -- -- NS HD NG NG NG HD HD HD HD NN HD NG HD NI HD NN N*	PXO421, PXO513, PXO364, PXO404
	-- -- -- -- -- -- -- -- -- -- -- -- -- NS HD NG HD NI HD NN N*	XM9
	-- -- -- NS HD NG NG HG NG HD HD NG HD NN HD NG HD NI NI NI N*	SK2-3, IX-280
TalDR	-- -- -- NS HD NG NG NG NG HD NN HD HD NN HD HD HD HD NN H*	HuN37
	-- -- -- NS HD NG NG NG NG HD HD HD HD NN HD HD HD HD NN H*	PXO563, PXO61, JL25, PXO421, PXO513, PXO404, K3, PXO364, JL28, PXO71, JL33, PXO142, K-74
	-- -- -- NS HD NG NG NG NG HD HD HD HD NN HD HD HD HD H*	K3a, AH28
	-- -- -- NS HD NG NG NG NG NG HD HD HD NN HD HD NG HD NI NN N*	CIAT

**TalDQ**	-- -- -- NS HD NG NG NG NG HD HD HD NN HD NG HD NI HD N*	PXO71
	-- -- -- -- -- -- -- -- -- -- -- -- NS HD NG NI NI NI N*	PXO524
**TalAW**	-- -- -- -- NS HD NG NG NG HD HD NG HD NN NG HD NN HD NG HD NI N*	PXO99, PXO79
**TalCM**	-- NS ND HG HG HG NG HG HG HD HD HD NN NN HD HG HH H* H* NN HD H*	BLS256, CFBP2286
	-- NI ND HG HG HG NG HG HG HD HD HD NN NN HD HG HH H* H* NN HD H*	BLS279, L8
	NI ND HG HG HG HG NG HG HG HD HD HD HD NN NN HD HG HH H* H* NN HD H*	B8-12, RS105
**TalCO**	-- -- -- NI HG HG HG HG NG HD HD HD HD HD NG N* NI NN HD HD H*	BXOR1, 0-9
**TalIB**	-- -- NI HG HG HG HG HG NG HD HD HD NN HD HH N* N* NI NN HD H*	GX01
	28 aa, RVD NG (p4); 37aa v3, RVD HD (p9)	
**TalHT**	-- -- -- NS HD NG NG NG NG HD HD NG HD NI HD NG HD NI NI N*	IXO704
	-- -- -- NS HD NG NG NG NG HD HD HD HD NN HD NG HD	AH28

All TALE classes with at least one member carrying an aberrant repeat are included. Only unique RVD sequences are listed, TALE classes and strains in which they occur are indicated. RVD sequences are grouped and aligned according to the aberrant repeat variant. Aberrant repeats are underlined. RVDs are coloured according to specificity (see Fig. 1). *X. oryzae* pv. *oryzae*, *Xoo*: IXO704, AUST2013, JW11089, KACC10331, KXO85, K1, K2, PXO61, PXO142, PXO404, PXO421, PXO513, PXO364, XM9, JP01, MAFF311018, PXO524, PXO83, PXO86, PXO145, PXO236, PXO2684, PXO1865, K3a, YC11, ScYc-b, JL33, JL25, JL28, PXO211, PXO563, PXO602, HuN37, XF89b, K-74, PXO71, IXO1088, PXO79, IX-280, SK2-3, PXO99, K3, ICMP3125, PXO282, CIAT, AH28, T7133, DY89031 (J18); *X. oryzae* pv. *oryzicola, Xoc*: CFBP2286, BLS279, GX01, 0-9, L8, BLS256, RS105, B8-12, BXOR1; *X. axonopodis* pv *citri, Xac*: Xac29-1; *X. citri* pv. *citri*, *Xcc*: Xcc49, 306, mf20, A306, LH201, jx5, UI7, FB19, LL074-4, MN11, MN10, jx4, LJ207-7, MN12, NT17, gd2, UI6, LH276, BL18, gd3, jx-6, 5208, 03-1638-1-1, Xcc29-1; *X. citri* pv. *punicae*, *Xcp*: LMG7439; *X.* sp: gxlp16; *X. theicola, Xth*: CFBP4691.

Table S3: Unique nucleotide and amino acid sequences of all known natural occurring aberrant repeats.

aberrant repeat	nucleotide and amino acid sequences	strain
	42 aa; RVD NI	
TalAQ	CTGACCCCGGAC------------------------**CAGGTGGTGGCCATCGCCAGTAAT**ATTGGCGGCAAGCAGGCGCTGGAGACGGTGCAGCTGCTGTTGCCGGTGCTGTGCCAGGACCATGG<br>L T P D **Q V V A I A S N** I G G K Q A L E T V Q L L L P V L C Q D H G	AUST2013, IX-280, JW11089, KXO85, SK2-3, IXO704, K1, K2
TalAQ	CTGACCCCGGAC------------------------**CAGGTGGTGGCCATCGCCAGTAAT**ATTGGCGGCAAGCAGGCGCTGGAGACGGTGCAGCGGCTGTTGCCGGTGCTGTGCCAGGACCATGG<br>L T P D **Q V V A I A S N** I G G K Q A L E T V Q R L L P V L C Q D H G	K-74, PXO71, PXO524, PXO602, XF89b, PXO404, PXO421, PXO513, PXO61
TalAQ	CTGACCCTGGCG------------------------**CAGGTGGTGGCCATCGCCAGCAAT**ATTGGCGGCAAGCAGGCGCTGGAGACGGTGCAGCGGCTGTTGCCGGTGCTGTGCCAGGACCATGGC<br>L T L A **Q V V A I A S N** I G G K Q A L E T V Q R L L P V L C Q D H G	T7133
TalAQ	CTGACCCCGGAC**CAGGTCGTGGCCATCGCCAGTAATCAGGTGGTGGCCATCGCCAGTAAT**ATTGGCGGCAAGCAGGCGCTGGAGACGGTGCAGCGGCTGTTGCCGGTGCTGTGCCAGGACCATGGC<br>L T P D **Q V V A I A S N Q V V A I A S N** I G G K Q A L E T V Q R L L P V L C Q D H G	PXO99A, MAFF311018, PXO83, PXO86, PXO211, PXO236, PXO563, ICMP3125, JL25, JL28, JP01, PXO79, ScYc-b, XM9, YC11, IXO1088, K3, K3a, AH28
TalAQ	CTGACCCCGGAC**CAGAGCGTGGCCATCGCCAGTAATCGGGTGGTGGCCATCGCCAGTAAT**ATTGGCGGCAAGCAGGCGCTGGAGACGGTGCAGCGGCTGTTGCCGGTGCTGTGCCAGGACCATGGC<br>L T P D **Q S V A I A S N R V V A I A S N** I G G K Q A L E T V Q R L L P V L C Q D H G	PXO145
	40 aa; RVD N*	
TalAC	CTGACCCCGGACCATGTTGTGGCTATCGCCAGCAAT---GGCGGCAAGCAGGCGCTGGAGACGGTG**CAGCGGCTGTTGCCGGTACAGCGGCTGTTGCCGGTG**CTGTGCCAGGACCATGGC<br>L T P D H V V A I A S N * G G K Q A L E T V **Q R L L P V Q R L L P V** L C Q D H G	KACC10331
TalAC / TalBH / TalDS / TalDV	CTGACCCCGGACCAGGTCGTGGCCATCGCCAGCAAT---GGCGGCAAGCAGGCGCTGGAGACGGTG**CAGCGGCTGTTGCCGGTACAGCGGCTGTTGCCGGTG**CTGTGCCAGGACCATGGC<br>L T P D Q V V A I A S N * G G K Q A L E T V **Q R L L P V Q R L L P V** L C Q D H G	MAFF311018, PXO1865, PXO2684, PXO83, PXO86, PXO145, PXO236, PXO524, AUST2013, JP01, JW11089, KXO85, K1, K2, IXO704, PXO211, PXO563, PXO602, HuN37, T7133, DY89031 (J18)
TalBH / TalFX	CTGACCCCGGACCAGGTCGTGGCCATCGCCAGCAAT---GGCGGCAAGCAGGCGCTGGAGACGGTG**CAGCGGCTGTTGCCTGTACAGCGGCTGTTGCCGGTG**CTGTGCCAGGACCATGGC<br>L T P D Q V V A I A S N * G G K Q A L E T V **Q R L L P V Q R L L P V** L C Q D H G	PXO61, PXO142, JL25, JL28, JL33, PXO364, PXO404, PXO421, PXO513, ScYc-b, YC11, K3a, XM9, AH28, K-74
TalEQ	CTGACCCCGGACCAGGTCGTGGCCATCGCCAGCAAT---GGCGGCAAGCAGGCGCTGGAGACGGTG**CAGCGGCTGTTGCCTGTACAGCGGCTGTTGCCGGTG**CTGTGCCAGGCCAATGGC<br>L T P D Q V V A I A S N * G G K Q A L E T V **Q R L L P V Q R L L P V** L C Q A N G	XF89b

	40 aa; RVD NN	
TalAS	CTGACCCCGGACCAAGTGGTGGCCATCGCCAACAATAACGGCGGCAAGCAGGCGCTGGAGACGGTG------------------**CAGCGGCTGTTGCCGGTG**CTGTGCCAGGACCATGGC<br>L T P D Q V V A I A N N N G G K Q A L E T V **Q R L L P V** L C Q D H G	IX-280, PXO79, SK2-3, IXO1088
	CTGACCCCGGACCAGGTGGTGGCCATCGCCAACAATAACGGCGGCAAGCAGGCGCTGGAGACGGTG------------------**CAGCGGCTGGTGCCGGTG**CTGTGCCAGGACCATGGC<br>L T P D Q V V A I A N N N G G K Q A L E T V **Q R L V P V** L C Q D H G	PXO211, PXO145, PXO86, PXO83
TalAS / TalER	CTGACCCCGGACCAGGTGGTGGCCATCGCCAACAATAACGGCGGCAAGCAGGCGCTGGAGACGGTG**CAGCGGCTGTTGCCGGTACAGCGGCTGGTGCCGGTG**CTGTGCCAGGACCATGGC<br>L T P D Q V V A I A N N N G G K Q A L E T V **Q R L L P V Q R L V P V** L C Q D H G	PXO142, PXO602, PXO563, PXO524, PXO71, KACC10331, MAFF311018, K-74, HuN37, JL33, JP01, JW11089, KXO85, PXO61, XM9, YC11, K1, K2, K3a, XF89b, AH28, T7133
TalAS	CTGACCCCGAACCAGGTGGTGGCCATCGCCAACAATAACGGCGGCAAGCAGGCGCTGGAGACGGTG**CAGCGGCTGTTGCCGGTACAGCGGCTGGTGCCGGTG**CTGTGCCAGGACCATGGC<br>L T P N Q V V A I A N N N G G K Q A L E T V **Q R L L P V Q R L V P V** L C Q D H G	AUST2013
	37 aa v1; RVD NG	
TalGB	CTGACCCCGGACCAGGTGGTGGCCATCGCCAGCAATGGCGGTGGCAAGCAGGCGCTGGAGACGGTGCAGCGGCTGTTGCCGGTGCTGTGCCAG**GCCCATGGCCCCCATGGC**<br>L T P D Q V V A I A S N G G G K Q A L E T V Q R L L P V L C Q **A H G P H G**	Xac29-1, Xcc49
	CTGACCCCGGACCAGGTGGTGGCCATCGCCAGCAATGGCGGTGGCAAGCAGGCGCTGGAGACGGTGCAGCGGCTGTTGCCGGTGCTGTGCCAG**GCCCATGGC**<br>L T P D Q V V A I A S N G G G K Q A L E T V Q R L L P V L C Q **A H G**	306, mf20, A306, LH201, jx5, UI7, FB19, LL074-4, MN11, MN10, jx4, LJ207-7, MN12, NT17, gd2, UI6, LH276, BL18, gd3, jx-6, 5208, gxlp16, 03-1638-1-1, Xcc29-1
	CTGACCCCGCAGCAGGTGGTGGCCATCGCCAGCAATGGCGGTGGCAAGCAGGCGCTGGAGACGGTGCAGCGGCTGTTGCCGGTGCTGTGCCAG**GCCCATGGC**<br>L T P Q Q V V A I A S N G G G K Q A L E T V Q R L L P V L C Q **A H G**	LMG7439
	37 aa v2; RVD HD	
TalGA	CTGACCCCGGAGCAGGTGGTGGCCATCGCCAGCCACGATGGCGGCAAGCAGGCGCTGGAGACG**GTGCAGCGGGTGCAGCGG**CTGTTGCCGGTGCTGTGCCAGGCCCATGGC<br>L T P E Q V V A I A S H D G G K Q A L E T **V Q R V Q R** L L P V L C Q A H G	Xac29-1
	CTGACCCCGGAGCAGGTGGTGGCCATCGCCAGCCACGATGGCGGCAAGCAGGCGCTGGAGACG**CTGCAGCGGGTGCAGCGG**CTGTTGCCGGTGCTGTGCCAGGCCCATGGC<br>L T P E Q V V A I A S H D G G K Q A L E T **L Q R V Q R** L L P V L C Q A H G	Xcc49
	37 aa v3; RVD HD	
TalHT	CTGACCCTGGACCAGGTAGTGGCCATTGCCAGCCAC**GATGGCGGCGATGGCGGC**AAGCAGGCGCTGGAGACGGTGCAGCGGCTGTTGCCGGTGCTGTGCCAGGCCCATGGT<br>L T L D Q V V A I A S H **D G G D G G** K Q A L E T V Q R L L P V L C Q A H G	AH28
	36 aa v1; RVD HD or NS	
TalFL	CTGACCCCGGACCAGGTCGTGGCCATCGCCAGCCACGATGGCGGCAAGCAGGCGCTGGAGACGGTGCAGCGCCTGTTG**CCGGTGCCGGTG**CTGTGCCAGGCCCATGGC<br>L T P D Q V V A I A S H D G G K Q A L E T V Q R L L **P V P V** L C Q A H G	AUST2013, IXO704
TalBK	CTGACCCCGGCCCAGGTCGTGGCCATCGCCAGCCACGATGGCGGCAAGCAGGCGCTGGAGACGGTGCAGCGCCTGTTG**CCGGTGCCGGTG**CTGTGCCAGGCCCATGGC<br>L T P A Q V V A I A S H D G G K Q A L E T V Q R L L **P V P V** L C Q A H G	PXO142, PXO61,
	CTGACCCCGGACCAGGTGGTGGCCATCGCCAGCAATAGTGGCGGCAAGCAGGCGCTGGAGACGGTGCAGCGCCTGTTG**CCGGTGCCGGTG**CTGTGCCAGGCCCATGGC<br>L T P D Q V V A I A S N S G G K Q A L E T V Q R L L **P V P V** L C Q A H G	PXO531, PXO404, PXO421, PXO364, XM9, PXO35, JW11089, K1, K2, KX085, KACC10331, DY89031 (J18)

	36 aa v2; RVD NI	
TalBV	CTGACCCCGGCGCAGGTGGTGGCCATCGCCAGCAATATTGGCGGCAAGCAGGCGCTGGAGACGGTGCAGCGGCTGTTGCCGGTGCTTTGCCAG**GCCCAT**------GGC<br>L T P A Q V V A I A S N I G G K Q A L E T V Q R L L P V L C Q **A H** G	BXOR1
	CTGACCCCGGCGCAGGTGGTGGCCATCGCCAGCAATATTGGCGGCAAGCAGGCGCTGGAGACGGTGCAGCGGCTGTTGCCGGTGCTGTGCCAG**GCCCAT**------GGC<br>L T P A Q V V A I A S N I G G K Q A L E T V Q R L L P V L C Q **A H** G	BLS256, CFBP2286, GX01, 0-9
	CTGACCCCGGCGCAGGTGGTGGCCATCGCCAGCAATATTGGCGGCAAGCAGGCGCTGGAGACGGTGCAGCGGCTGTTGCCGGTGCTGTGCCAG**GCCCATGCCCAT**GGC<br>L T P A Q V V A I A S N I G G K Q A L E T V Q R L L P V L C Q **A H A H** G	B8-12, BLS279, L8, RS105
	36 aa v3; RVD NI	
TalHR	CTGACCCCGGCGCAGGTCGTGGGCATCGCCAGCAACATCGGC**GGCGGC**AAGCAGGCGCTGGAGACGGTGCAGCGGCTGCTGCCCGAGTTGTGCAAGCCCCCCTATGGC<br>L T P A Q V V G I A S N I G **G G** K Q A L E T V Q R L L P E L C K P P Y G	CFBP4691
	30 aa; RVD NI or NN	
TalBG	CTGATCCCGGACCAGGTGGTGGCCATCGCCAGCAATATTGGCGGCAAGCAGGCGCTGGAGACGGTGCAGCGGCTGTTGCCGGTGCTGTGC------------<br>L I P D Q V V A I A S N I G G K Q A L E T V Q R L L P V L C	BLS256, CFBP2286
	CTGACCCCAGACCAGGTGGTGGCCATCGCCAGCAATAACGGCGGCAAGCAGGCGCTGGAGACGGTGCAGCGGCTGTTGCCGGTGCTGTGC------------<br>L T P D Q V V A I A S N N G G K Q A L E T V Q R L L P V L C	B8-12, BLS279, L8, RS105
	28 aa; RVD NG or HG	
TalCM	CTGACCCCGGACCAGGTGGTGGCCATCGCCAGCAATGGCGGCGGCAAGCAGGCGCTGGAGACG------------------GTACTGCGCCAAGCCCATGGC<br>M T P D Q V V A I A S N G G G K Q A L E T V L R Q A H G	B8-12, BLS279, CFBP2286, L8, RS105,, BLS256
TalAW	CTGACCCCGGACCAGGTGGTGGCCATCGCCAGCAATGGCGGCGGCAAGCAGGCGCTGGAGACG------------------GTACTGTGTCAGGCCCATGGC<br>M T P D Q V V A I A S N G G G K Q A L E T V L C Q A H G	PXO79, PXO99A
TalAI / TalDR / TalDQ / TalHT	CTGACCCCGGACCAGGTGGTGGCCATCGCCAGCAATGGCGGCGGCAAGCAGGCGCTGGAGACG------------------GTACTGTGCCAGGCCCATGGC<br>M T P D Q V V A I A S N G G G K Q A L E T V L C Q A H G	PXO99, MAFF311018, PXO83, PXO86, PXO145, PXO211, PXO236, PXO282, PXO524, PXO563, PXO602, XF89b, ICMP3125, PXO142, IX-280, JP01, PXO364, PXO404, PXO421, PXO513, PXO61, PXO79, ScYc-b, SK2-3, XM9, YC11, IXO11088, K3, K3a, K-74, PXO71, CIAT, HuN37, JL25, JL28, JL33, AH28, T7133
TalCO / TalIB	CTGACCCCGGACCAGGTGGTGGCCATCGCCAGCCATGGCGGCGGCAAGCAGGCGCTGGAGACG------------------GTGCTGTGCCAGGCCCATGGC<br>M T P D Q V V A I A S H G G G K Q A L E T V L C Q A H G	0-9, BXOR1, GX01

*Nucleotides encoding RVDs and the RVDs are highlighted in yellow, differences between naturally occurring variants of an aberrant repeat type are highlighted in green. If the TALE class carrying an aberrant repeat has members without the aberrant repeat, the 34 aa repeat from the corresponding position in these TALEs was included. Regions responsible for the length alteration are indicated in bold. Normal and dashed lines highlight the regions duplicated or deleted.

Table S4: Aberrant repeat modules generated or used in this study.

aberrant repeat	repeat position[1]					
	RVD	1	2	3	4	5	6
42 aa	HD	+	+	+	+	+	+
	NN	+	+	+	+	+	+
	NI	+ / s	+ / s	+ / s	+ / s	+ / s	+
	NG	+	+	+	+	n/c	+
40 aa	HD	+	+	+	+	+	(+)
	NN	+	+	+	+	+	(+)
	NI	+	+	+	+	+	(+)
	NG	+	+	+	+	+	(+)
	N*	n/c	+	+	n/c	n/c	(+)
37 aa v1	HD	+	+	+	+	n/a	n/a
	NN	+	+	+	+	n/a	n/a
37 aa v2	HD	+	+	+	+	n/c	n/a
	NN	+	+	n/c	+	n/c	n/a
36 aa v1	HD	n/c	+	+	n/c	n/c	n/a
	NN	n/c	+	+	n/c	n/c	n/a
	NI	+	+	+	n/c	n/c	n/a
	NS	+	+	n/c	n/c	n/c	n/a
36 aa v2	HD	n/c	+	n/c	n/c	n/c	n/a
	NN	n/c	+	n/c	n/c	n/c	n/a
	NI	n/c	+	n/c	n/c	n/c	n/a
36 aa v3	HD	n/c	+	n/c	n/c	n/c	n/a
30 aa	HD	+	+	+	+	n/a	n/a
	NN	+	+	+	+	n/a	n/a
	NI	+	+	+	+	n/a	n/a
	NG	+	+	+	+	n/a	n/a
28 aa	HD	n/c	+	+	n/c	n/c	n/a
	NN	+	+	+	n/c	n/c	n/a
	NI	n/c	+	+	n/c	n/c	n/a
	NG	+	+	+	n/c	n/c	n/a

[1] All generated modules are fully compatible with the Golden TALE assembly kit (Geißler *et al.*, 2011 PLoS One, 6, e19509). A ”+” indicates the successful construction of this repeat module, a “+/s” indicates that a normal repeat and as well as a stop-re peat were generated, “(+)” indicates that this RVD can be used in some cases but that a normal repeat with this RVD has to be combined with a special assembly vector. “n/c” indicates that the repeat module was not constructed and “n/a” indicates that the construction of this repeat module is not possible due to incompatibility between the Golden TALE assembly kit and the nature of the aberrant repeat.
